# A stochastic approximation expectation maximization algorithm for estimating Ramsay-curve three-parameter normal ogive model with non-normal latent trait distributions

**DOI:** 10.3389/fpsyg.2022.971126

**Published:** 2022-11-24

**Authors:** Yuzheng Cui, Jing Lu, Jiwei Zhang, Ningzhong Shi, Jia Liu, Xiangbin Meng

**Affiliations:** ^1^Key Laboratory of Applied Statistics of Ministry of Education, School of Mathematics and Statistics, Northeast Normal University, Changchun, China; ^2^Faculty of Education, Northeast Normal University, Changchun, China

**Keywords:** item response theory, Ramsay curve, 3PNO model, marginal maximum likelihood estimation, stochastic approximation EM algorithm (SAEM), density estimation

## Abstract

In the estimation of item response models, the normality of latent traits is frequently assumed. However, this assumption may be untenable in real testing. In contrast to the conventional three-parameter normal ogive (3PNO) model, a 3PNO model incorporating Ramsay-curve item response theory (RC-IRT), denoted as the RC-3PNO model, allows for flexible latent trait distributions. We propose a stochastic approximation expectation maximization (SAEM) algorithm to estimate the RC-3PNO model with non-normal latent trait distributions. The simulation studies of this work reveal that the SAEM algorithm produces more accurate item parameters for the RC-3PNO model than those of the 3PNO model, especially when the latent density is not normal, such as in the cases of a skewed or bimodal distribution. Three model selection criteria are used to select the optimal number of knots and the degree of the B-spline functions in the RC-3PNO model. A real data set from the PISA 2018 test is used to demonstrate the application of the proposed algorithm.

## 1. Introduction

A premise of item response theory (IRT) is that observed item responses are indicators of one or more latent traits. For item parameter estimation, the latent variable is usually presumed to be normally distributed. However, many psychological constructs, such as ambition, dysthymia, and borderline personality disorder, as well as other latent traits in sociology such as drug abuse, are unlikely to be normally distributed in a general population. For example, a psychiatric disorder trait is typically positively skewed in a general population because most people are located at the non-pathological end of the trait, whereas a small group of individuals is spread out along the mild, moderate, and severe end of the disorder (Woods, 2006; Woods and Thissen, 2006; Wall et al., 2015; Wang et al., 2018). In addition, variables representing symptoms of pathology that are rare in the general population may be skewed because they exist at low levels for most people and at high levels for a few individuals. Therefore, the assumption of the normal distribution of latent traits leads to biased parameter estimates when the true latent trait distribution *g*(θ) is non-normal (Woods, 2006, 2007; Woods and Thissen, 2006; Woods and Lin, 2009; Azevedo et al., 2011; DeMars, 2012; Molenaar et al., 2012; Reise and Revicki, 2014; Wall et al., 2015; Reise et al., 2018).

Various studies have developed approaches to dealing with non-normal distributions of latent traits. In particular, the empirical histogram (EH) approach (Bock and Aitkin, 1981; Reise and Revicki, 2014) has been proposed to estimate the height of the latent trait density *g*(θ) at each quadrature point (i.e., the values on the θ continuum, usually the number of quadrature points is large) instead of computing the heights based on the normal density. This is more flexible than the expectation maximization (EM) algorithm (Bock and Lieberman, 1970; Dempster et al., 1977; Bock and Aitkin, 1981), which is restricted to the normal assumption of latent traits. However, the EH method is sensitive to the user-specified rectangular quadrature scheme. In addition, the graphical representations from the EH method are usually “choppy” or jagged, which makes it difficult to use them to clarify the characterizations of latent traits.

To address this issue, several methods have been proposed to approximate the latent trait density more precisely, including log-linear smoothing (LLS; Casabianca and Lewis, 2015), the Davidian curve (Woods and Lin, 2009), and the Ramsay curve (Woods, 2006; Woods and Thissen, 2006). When incorporated with IRT, these methods simultaneously estimate the latent trait density and the item parameters, but they use different approaches to estimate the latent trait density *g*(θ). Specifically, LLS matches *M* moments of the original distribution to create a smoothed distribution of latent traits while making fewer assumptions about its form and maintaining parsimony. Davidian-curve IRT (DC-IRT; Woods and Lin, 2009) provides a smooth representation of *g*(θ) using a unidimensional Davidian curve, as described by Zhang and Davidian (2001). Ramsay-curve IRT (RC-IRT; Woods, 2006; Woods and Thissen, 2006) estimates the latent trait density using Ramsay curves based on B-spline functions.

This paper focuses on RC-IRT because it is one of the most flexible and easy-to-apply methods for estimating the latent-ability density. Woods and Thissen (2006) first introduced RC-IRT to detect and correct for the non-normality of *g*(θ), and they estimated the item parameters of the two-parameter logistic (2PL; Birnbaum, 1968) model using the marginal maximum likelihood estimation with the EM (MML-EM; Baker and Kim, 2004) method. Newton–Raphson iteration was used to update the shape parameter ***η***. Woods (2008) extended this approach to estimate an RC-IRT model for the three-parameter logistic (3PL; Birnbaum, 1968) model. Subsequently, Monroe and Cai (2014) proposed using a Metropolis–Hastings–Robbins–Monro (MHRM; Cai, 2010) algorithm to estimate an RC-IRT approach for the unidimensional graded response model (GRM; Samejima, 1969). The major advantage of this approach is that the covariance matrix estimates can be easily obtained as a byproduct. However, in their study, the GRM was limited to the logistic version.

To date, there have been no studies examining RC-IRT in the context of the normal ogive model. The reason for this is that the EM algorithm usually involves numerical integration calculations, which is intractable for the normal ogive model itself because it already includes an integral term. To fill this knowledge gap, we propose to estimate the RC-3PNO model using a stochastic approximation EM (SAEM; Delyon et al., 1999) algorithm. Specifically, the integral calculation in the E-step is replaced by a stochastic approximation method, which greatly simplifies the calculation. After introducing latent variables, the complete data likelihood can be transformed into an exponential family distribution; thus, sufficient statistics can be used to easily update the estimates of item parameters in the M-step. This avoids the calculation of derivatives in the original M-step in the EM algorithm and further improves the computation efficiency. The latent density distribution *g*(θ) is estimated using traditional Newton–Raphson iteration for the proposed SAEM algorithm.

Note that for the estimation of shape parameter ***η*** and item parameters, the Bayesian maximum a posteriori (MAP; Greig et al., 1989) estimate is used (this will be interpreted further in Section 3). The posterior simulation methods make the posterior distributions easier to obtain; that is, the algorithms for posterior simulation can be used to obtain approximates of posterior moments. Various Bayesian estimates can be obtained based on the posterior samples obtained from the posterior distributions. In this study, two Bayesian estimates are used. One method is MAP estimate. In fact, the MAP estimate is an estimate of an unknown quantity, which is equal to the mode of the posterior distribution. The MAP can be used to obtain point estimates of an unobserved quantities based on empirical data. It is closely related to the maximum likelihood estimate, but employs an augmented optimization objective that incorporates a prior distribution (additional information available by quantifying the prior knowledge of the interested events). Therefore, MAP estimate can be seen as a regularization of maximum likelihood estimate. Another method is marginal maximum a posteriori (MMAP; Mislevy, 1986; Lee and Gauvain, 1996; Baker and Kim, 2004) estimate. MMAP estimate can be seen as an extension of the MAP estimate by integrating out the latent variables as the nuisance parameters and then obtaining MAP estimates of the interested parameters. More details for estimation forms of our model can be found in Sections 3 and 4.

The remainder of this article is organized as follows. The second section presents the 3PNO model incorporating a Ramsay curve (denoted as the RC-3PNO model). The third section gives the marginal maximum a posteriori (MMAP) estimation of all the parameters to be estimated in the RC-3PNO model. The fourth section introduces the SAEM procedure for estimating the RC-3PNO model (hereafter referred to as the RC-SAEM algorithm), which is the main contribution of this study. The fifth section presents two simulation studies: one to select the optimal number of knots and the appropriate degree of the B-spline functions for the Ramsay curve and another to assess the performance of the proposed algorithm. A real data set from the PISA 2018 test is then used to demonstrate the application of the proposed algorithm in the sixth section. Finally, conclusions and directions for future research are provided.

## 2. The RC-3PNO model

Let *U*_*ij*_ (with realization *u*_*ij*_) denote the dichotomous response variable of examinee *i* (*i* = 1, ⋯ , *N*) to item *j* (*j* = 1, ⋯ , *J*); *U*_*ij*_ = 1 denotes the correct response, and *U*_*ij*_ = 0 otherwise. The 3PNO model is defined as

(1) $$\begin{array}{l} P_{j}\left(\theta_{i}\right)=P\left(U_{ij}=1|\Omega_{j},\theta_{i}\right)=c_{j}+\left(1-c_{j}\right)\Phi \left(a_{j}\theta_{i}+b_{j}\right), \end{array}$$

where: Φ(·) is the cumulative function of the standard normal distribution; **Ω**_*j*_ = (*a*_*j*_, *b*_*j*_, *c*_*j*_) denotes the characteristic parameters of item *j*, in which *a*_*j*_ ∈ [0, +∞) is the discrimination parameter, *b*_*j*_ ∈ (−∞, +∞) is the intercept parameter, and *c*_*j*_ ∈ [0, 1] is the guessing parameter; and θ_*i*_ ∈ (−∞, +∞) is the latent-ability parameter of examinee *i*.

The latent trait distribution should be given in advance for the use of the MML and MMAP estimations in IRT models. In general, it is assumed that θ_*i*_ ~ *N*(0, 1), which is convenient for statistical computation. However, the normal-distribution assumption is not likely to be tenable, and this will decrease the accuracy of estimates from the MML and MMAP estimations. To address this issue, Woods and Thissen (2006) proposed the Ramsay-curve IRT, which is based on B-spline functions, to describe the latent trait distribution. This provides greater flexibility than the standard normal distribution of latent traits.

Following Woods and Thissen (2006), the latent trait distribution is modeled by

(2) $$\begin{array}{l} g\left(x_{q}|\eta \right)=\frac{\exp\left[B^{*}\left(x_{q}\right)\eta \right]}{C}, \end{array}$$

where

(3) $$\begin{array}{l} C=\overset{Q}{\underset{q=1}{\sum }}\exp\left[B^{*}\left(x_{q}\right)\right] \end{array}$$

is the normalization constant ensuring that *g*(*x*_*q*_|***η***) (*q* = 1, 2, ⋯ , *Q*) sums to 1. In this study, *x*_*q*_ represents the 121 fixed points with equidistant distance along the interval [-6,6]. To avoid ambiguity with the latent variable with continuous support, the discrete *x*_*q*_ is used here.

In RC-IRT, the density of latent ability, *g*(θ), needs to be estimated simultaneously with the item parameters. The shape of the latent-ability density curve is determined by a shape parameter ***η***, which is a vector whose dimensionality is controlled by the choice of *knots* and *degree*. The dimension of ***η*** is *m* = *degree* + *knots* − 1. The support of the Ramsay-curve density can be numerically represented over a set of discrete points {*x*_*q*_ : *q* = 1, 2, ⋯ , *Q*} along the real number line. In this study, the discrete points are fixed at 121 equidistant values from −6 to 6 separated by steps of 0.1. The interval [−6, 6] is a range of latent traits often used in RC-IRT (Woods and Thissen, 2006; Monroe and Cai, 2014), and this can contain the great majority of the latent abilities being tested. The *knots* are values at which the B-spline functions are connected to each other. Typically, the *knots* are evenly distributed over the range of θ. The number of *knots* in RC-IRT is usually selected from the range 2 to 6 (Woods and Thissen, 2006; Monroe and Cai, 2014). The parameter *degree* refers to the degree of the basic B-spline function. To some extent, although a larger number of *knots* may create a more flexible estimated density curve and a higher *degree* value could accommodate a sharper curve. However, sometimes the increase of *knots* or *degree* will make the Ramsay curve become overfitted, resulting in a more complex model than the appropriate model. And this will increase the parameter to be estimated in the model, which may deteriorate the estimation.

Given *knots*, *degree*, and the discrete points *x*_*q*_, the corresponding basic B-spline functions $B^{*}\left(x_{q}\right)$ are determined. The definition and derivation of B-spline function are beyond the scope of this study, and the interested readers can consult De Boor (1978) for details. Gathering *Q* discrete points together, the basic B-spline functions can be expressed as a *Q* × *m* matrix B^*^. The matrix B^*^ is assumed to be known here, and the only parameter that needs to be estimated is ***η***. Equations (1) and (2) construct the RC-3PNO model, which incorporates the Ramsay curve into the 3PNO model.

## 3. MMAP estimation for the RC-3PNO model

We denote Θ = (θ_1_, θ_2_, ⋯ , θ_*N*_) and let **Ω** = **(a, b, c)**, where **(a, b, c)** = (*a*_*j*_, *b*_*j*_, *c*_*j*_) (*j* = 1, 2, ⋯ , *J*). The parameters of the RC-3PNO model that need to be estimated are **Ω** and ***η***, denoted as ***ζ*** = (**Ω**, ***η***).

The conditional distribution of *U*_*ij*_ given **Ω** and Θ has a binomial form:

(4) $$\begin{array}{l} f\left(u_{ij}|\theta_{i},\Omega_{j}\right)={P_{j}\left(\theta_{i}\right)}^{u_{ij}}{\left[1-P_{j}\left(\theta_{i}\right)\right]}^{1-u_{ij}}, \end{array}$$

where *P*_*j*_(θ_*i*_) is equal to Equation (1). Based on the local conditional independence assumption (Birnbaum, 1968), the probability of examinee *i*'s conditional response pattern is

(5) $$\begin{array}{l} f\left(u_{i}|\theta_{i},\Omega_{j}\right)=\overset{J}{\underset{j=1}{\prod }}f\left(u_{ij}|\theta_{i},\Omega_{j}\right). \end{array}$$

The observed data is response matrix **U**, the person parameter Θ = {θ_1_, θ_2_, ⋯ , θ_*N*_} is viewed as the missing data, and thus the complete data is (**U**, Θ). Conditional independence of item responses is assumed, as well as the independence of respondents, in accordance with practice. Thus, the complete data likelihood of (**U**, Θ) is

(6) $$\begin{array}{l} L\left(\zeta |\text{U},\Theta \right)=\overset{N}{\underset{i=1}{\prod }}\overset{J}{\underset{j=1}{\prod }}{P_{j}\left(\theta_{i}\right)}^{u_{ij}}{\left[1-P_{j}\left(\theta_{i}\right)\right]}^{1-u_{ij}}g\left(\theta_{i}|\eta \right), \end{array}$$

where *P*_*j*_(θ_*i*_) is equal to Equation (1). Taking the natural logarithm of Equation (6), the log-likelihood of (**U**, Θ) can be expressed as

(7) $$\begin{array}{l} \log L\left(\zeta |\text{U},\Theta \right)=\log L\left(\Omega |\text{U},\Theta \right)+\log L\left(\eta |\Theta \right), \end{array}$$

where

(8) $$\begin{array}{l} \log L\left(\Omega |\text{U},\Theta \right)=\overset{N}{\underset{i=1}{\sum }}\overset{J}{\underset{j=1}{\sum }}\left\{u_{ij}\log\frac{P_{j}\left(\theta_{i}\right)}{1-P_{j}\left(\theta_{i}\right)}+\log\left[1-P_{j}\left(\theta_{i}\right)\right]\right\} \end{array}$$

and

(9) $$\begin{array}{l} \log L\left(\eta |\Theta \right)=\overset{N}{\underset{i=1}{\sum }}\log g\left(\theta_{i}|\eta \right). \end{array}$$

The complete data log-likelihood in Equation (7) can be seen as the sum of two independent parts: the logarithm of the likelihood of item parameters **Ω** and the logarithm of the likelihood of ***η***. Thus, the processes of estimating **Ω** and ***η*** can be conducted separately (Monroe and Cai, 2014), which improves the computational efficiency. Here, the MMAP estimation of ***ζ*** = (**Ω**, ***η***) is given in detail. The priors of **Ω** are given below. The prior distribution for (*a*_*j*_, *b*_*j*_) is specified as

(10) $$\begin{array}{l} {\left(a_{j},b_{j}\right)}^{\prime }\sim N_{2}\left(\mu ,\Sigma \right)I\left(a_{j}>0\right) \end{array}$$

for *j* = 1, 2, ⋯ , *J*, where *N*_2_(·) denotes a bivariate normal distribution. The prior distribution for *c*_*j*_ is chosen to be,

(11) $$\begin{array}{l} c_{j}\sim \text{Beta}\left(\alpha ,\beta \right) \end{array}$$

for *j* = 1, 2, ⋯ , *J*. According to previous methods of estimating ***η*** in RC-IRT (Woods and Thissen, 2006; Monroe and Cai, 2014), a diffuse prior density of ***η*** is assumed:

(12) $$\begin{array}{l} \eta \sim \text{MVN}\left(\mu_{\eta },\Sigma_{\eta }\right). \end{array}$$

The estimation of ***η*** will be introduced in detail later.

From Equation (6), the marginalized likelihood of ***ζ*** is

(13) $$\begin{array}{l} L\left(\zeta |\text{U}\right)=\overset{N}{\underset{i=1}{\prod }}\left\{\int \overset{J}{\underset{j=1}{\prod }}{P_{j}\left(\theta_{i}\right)}^{u_{ij}}{\left[1-P_{j}\left(\theta_{i}\right)\right]}^{1-u_{ij}}g\left(\theta_{i}|\eta \right)d\theta_{i}\right\}\; \end{array}$$

Based on the priors in Equations (10)–(12) and the marginalized likelihood in Equation (13), the marginalized posterior distribution of ***ζ*** = (**Ω**, ***η***) is

(14) $$\begin{array}{l} f\left(\zeta |\text{U}\right)=f\left(\Omega ,\eta |\text{U}\right)\\ =\frac{L\left(\Omega ,\eta |\text{U}\right)f\left(\Omega \right)f\left(\eta \right)}{\int \int L\left(\Omega ,\eta |\text{U}\right)f\left(\Omega \right)f\left(\eta \right)d\Omega d\eta }\propto L\left(\Omega ,\eta |\text{U}\right)f\left(\Omega \right)f\left(\eta \right), \end{array}$$

where

(15) $$\begin{array}{l} f\left(\Omega \right)=\overset{J}{\underset{j=1}{\prod }}f\left(a_{j},b_{j}\right)f\left(c_{j}\right)I\left(a_{j}>0\right) \end{array}$$

is the joint prior density function of **Ω**, and *f*(***η***) denotes the density function of a multivariate normal distribution. Thus, the MMAP estimation of ***ζ*** = (**Ω**, ***η***) is

(16) $$\begin{array}{l} \hat{\zeta }=\underset{\zeta \in \Theta_{\zeta }}{\arg\max}\log f\left(\zeta |\text{U}\right)]=\arg\max[\log L\left(\zeta |\text{U}\right)\\ +\log f\left(\Omega \right)+\log f\left(\eta \right)]. \end{array}$$

In the next section, an SAEM algorithm is developed to compute the MMAP estimate of ***ζ*** in Equation (16), which is the main contribution of this study. Note that the SAEM algorithm includes a stochastic approximation step instead of the integral step in the EM algorithm (please see the next section for details); in other words, the SAEM algorithm does not need to marginalize the latent abilities, and the estimate of **Ω** in the SAEM algorithm thus belongs to the maximum a posteriori (MAP) estimate. In addition, we use Newton–Raphson iteration to obtain the MAP estimate of the shape parameter ***η***, which was also adopted by Woods and Thissen (2006).

## 4. SAEM algorithm for the RC-3PNO model

In this section, an SAEM algorithm for the RC-3PNO model is developed to compute the MAP estimates for the shape parameters of the Ramsay curve and the item parameters. First, the relationship between the standard EM algorithm and the SAEM algorithm is given. Second, a data-augmentation scheme for the 3PNO model is introduced, which means that the complete data likelihood formulation of the 3PNO model belongs to an exponential family form (Camilli and Geis, 2019; Geis, 2019). Sufficient statistics of the item parameters are also computed. Third, the estimation of the density curve of latent ability is depicted. Fourth, the estimation procedure of the SAEM algorithm is given for the RC-3PNO model.

### 4.1. Relationship between the EM algorithm and SAEM algorithm

The EM algorithm is briefly reviewed first. It is widely used in maximum likelihood or maximum a posteriori estimation for the incomplete data. The EM algorithm uses an expectation step (E-step) and a maximization step (M-step) to iteratively maximize the conditional expectation of the complete log-likelihood. In the E-step, the conditional expectation of the logarithmic complete data likelihood is adopted considering the observed data and the parameter values obtained in the previous step. In the M-step, the MAP estimates of the parameters are calculated based on the updated expectations in the E-step. The procedure alternates between E-step and M-step until convergence. In the case of exponential family distribution, the E-step and M-step can be simplified to update the expectation of the sufficient statistic and calculate the MAP estimate using the updated sufficient statistic, respectively.

However, in some cases, the EM algorithm is not applicable when either E-step or M-step is intractable or even cannot be performed in closed form. A possible solution for the complex computation of M-step is to replace the global optimization with a simpler conditional maximization chain, leading to the so-called ECM algorithm (Meng and Rubin, 1993). In IRT, the number of quadrature points in the numerical integral grows exponentially with the increasement of latent trait dimension. Therefore, the E-step will become very time-consuming as the latent trait dimension increases. Wei and Tanner (1990) proposed Monte Carlo EM (MCEM) to deal with this problem. The basic idea is to compute the expectation in the E-step by the Monte Carlo method. Geyer (1994) proved the convergence of the Monte Carlo maximum likelihood calculations, which provided the theoretical basis for MCEM. Delyon et al. (1999) proposed the SAEM algorithm as an alternative to the MCEM algorithm, which replaces the E-step of the EM algorithm with one iteration of the stochastic approximation procedure. Thereafter, the SAEM algorithm was widely used for its efficiency and convenience.

### 4.2. Data-augmentation scheme for the 3PNO model

The 3PNO model in Equation (1) can be rewritten as

(17) $$\begin{array}{l} P\left(U_{ij}=1|a_{j},b_{j},c_{j},\theta_{i}\right)=c_{j}\left(1-\Phi \left(a_{j}\theta_{i}+b_{j}\right)\right)+\Phi \left(a_{j}\theta_{i}+b_{j}\right), \end{array}$$

and its form can be seen as a mixture of two Bernoulli distributions with the categorical probability Φ(*a*_*j*_θ_*i*_ + *b*_*j*_). A dichotomous latent variable *W*_*ij*_ is defined, and *W*_*ij*_ = 1 denotes examinee *i* knowing the answer of item *j*, and *W*_*ij*_ = 0 otherwise. Because the 3PNO model does not contain a slipping parameter, examinees can answer the item correctly with probability 1 if they know the answer. The 3PNO model also includes a guessing parameter, and examinees can guess the correct answer with probability *c*_*j*_ if they do not know it. Thus, the following two equations hold:

(18) $$\begin{array}{l} P\left(U_{ij}|W_{ij}=1\right)=1^{U_{ij}}0^{1-U_{ij}}, \end{array}$$

(19) $$\begin{array}{l} P\left(U_{ij}|W_{ij}=0\right)=c_{j}^{U_{ij}}{\left(1-c_{j}\right)}^{1-U_{ij}}. \end{array}$$

A continuous latent variable *Z*_*ij*_ is then introduced:

(20) $$\begin{array}{l} W_{ij}=I\left(Z_{ij}>0\right), \end{array}$$

where *I*(·) denotes the indicator function. The following conditional distribution holds:

(21) $$\begin{array}{l} Z_{ij}|\theta_{i},a_{j},b_{j}\sim N\left(a_{j}\theta_{i}+b_{j},1\right). \end{array}$$

The conditional probability of *W*_*ij*_ is

(22) $$\begin{array}{l} P\left(W_{ij}=1|\theta_{i},a_{j},b_{j}\right)=P\left(Z_{ij}>0|\theta_{i},a_{j},b_{j}\right)=\Phi \left(a_{j}\theta_{i}+b_{j}\right). \end{array}$$

According to the total probability formula, the marginal probability of *U*_*ij*_ = 1 is

(23) $$\begin{array}{l} P\left(U_{ij}=1|\Omega_{j}\right)=\Phi \left(a_{j}\theta_{i}+b_{j}\right)+\left[1-\Phi \left(a_{j}\theta_{i}+b_{j}\right)\right]\times c_{j}, \end{array}$$

which is exactly the 3PNO model in Equation (1).

#### 4.2.1. Complete data likelihood of the 3PNO model

According to Equations (18)–(21), the joint distribution of (***U***, ***W***, ***Z***) is

(24) $$\begin{array}{l} P\left(U_{ij},W_{ij},Z_{ij}|\theta_{i},\Omega_{j}\right)=P\left(U_{ij}|W_{ij},\theta_{i},\Omega_{j}\right)\cdot P\left(W_{ij},Z_{ij}|\theta_{i},\Omega_{j}\right), \end{array}$$

where

(25) $$\begin{array}{l} P\left(U_{ij}|W_{ij},\theta_{i},\Omega_{j}\right)={\left[1^{W_{ij}}{c_{j}}^{1-W_{ij}}\right]}^{U_{ij}}\\ \cdot {\left[0^{W_{ij}}{\left(1-c_{j}\right)}^{1-W_{ij}}\right]}^{1-U_{ij}}, \end{array}$$

(26) $$\begin{array}{c} P\left(W_{ij},Z_{ij}|\theta_{i},\Omega_{j}\right)\\ =\left[I\left(W_{ij}=1\right)I\left(Z_{ij}>0\right)+I\left(W_{ij}=0\right)I\left(Z_{ij}<0\right)\right]\\ \times \phi \left(z_{ij}-a_{j}\theta_{i}-b_{j}\right). \end{array}$$

Let **z** and **w** denote the observations of augmented variables ***Z*** and ***W***, respectively. **Θ** denotes the data set of Θ sampled in the S1-step of SAEM (more details are given in a later subsection). Note that **Θ** is also the augmented data sets in SAEM, and the estimate of **Ω** is an MAP estimate. The augmented data sets are **Ψ** = (**Θ**, **w**, **z**), and the observed responses are **U**. The complete data likelihood of **Ω** can then be expressed as

(27) $$\begin{array}{l} L\left(\Omega |\text{U},\Psi \right)=\overset{N}{\underset{i=1}{\prod }}\overset{J}{\underset{j=1}{\prod }}P\left(U_{ij},W_{ij},Z_{ij}|\theta_{i},\Omega_{j}\right)g\left(\theta_{i}\right), \end{array}$$

According to Equations (24)–(26), we have

(28) $$\begin{array}{l} L\left(\Omega |\text{U},\Psi \right)\propto \prod_{j=1}^{J}\left\{c_{j}^{\sum_{i=1}^{N}\left(1-w_{ij}\right)u_{ij}}{\left(1-c_{j}\right)}^{\sum_{i=1}^{N}\left(1-w_{ij}\right)\left(1-u_{ij}\right)}\right\}\\ \;\times \prod_{i=1}^{N}\prod_{j=1}^{J}\left\{\phi \left(z_{ij}-a_{j}\theta_{i}-b_{j}\right)g\left(\theta_{i}\right)\right\}. \end{array}$$

It can be proved that *L*(**Ω**|**U**, **Ψ**) has a form of an exponential family distribution.

Two advantages need to be mentioned here. First, after introducing the augmented latent variables, the complete data likelihood has a form of exponential family distribution. Second, given complete data, the MAP estimates of item parameter **Ω** can be expressed as several functions that are only concerned with sufficient statistics. In this case, we can directly implement the computation of the MAP estimates of the item parameters that only need to update the sufficient statistics, which greatly reduces the computational complexity and improves the computational efficiency. In addition, the SAEM algorithm converges to the local maximum (Delyon et al., 1999). Note that, due to the data-augmentation scheme, Equation (16) is not the objective function to be optimized in the SAEM algorithm. Instead, **Θ** is viewed as augmented data in the SAEM algorithm and is thus included in the objective function to be optimized [see Equation (28)]. The augmented data of **Θ** can be used in the estimation of ***η*** (this is elaborated later).

### 4.3. Estimation of density curve of latent ability

The estimation of the non-normal ability density curve relies on the computation of the shape parameter ***η***, which involves an optimization algorithm using either Newton–Raphson iterations (Woods and Thissen, 2006) or an MHRM algorithm (Monroe and Cai, 2014). Once the estimates of ***η*** have been obtained, these estimates can be used in Equation (2) to calculate *g*(θ_*i*_|***η***) for a particular examinee or to construct the entire Ramsay-curve density. In this study, the Newton–Raphson iteration method was used to estimate ***η***, and the log-likelihood of the Ramsay curve is the objective function to be optimized. Its form is

(29) $$\begin{array}{l} \log L\left(\eta |\Theta \right)=\overset{N}{\underset{i=1}{\sum }}\log\left(g\left(\theta_{i}|\eta \right)\right). \end{array}$$

In practice, there may be some regions of the latent trait scale over which little or no information about the RC parameters ***η*** is available. As a result, the corresponding spline coefficients may become empirically underidentified (Woods and Thissen, 2006; Monroe and Cai, 2014). When this happens, the estimation of the entire set of coefficients will fail. To prevent such an estimation failure, a diffuse prior density is often assumed on ***η*** (Woods and Thissen, 2006; Monroe and Cai, 2014). The Bayesian MAP estimation can be used, and the Ramsay-curve posterior (RCP) density is then the product of the Ramsay-curve likelihood and an *m*-variate normal prior (Woods and Thissen, 2006; Monroe and Cai, 2014), where *m* is the number of coefficients. Since the normalization constant of the posterior is omitted, the logarithm RCP of ***η*** is given by

(30) $$\begin{array}{l} l_{R}\left(\eta |\Theta \right)\propto \log L\left(\eta |\Theta \right)-\frac{1}{2}{\left(\eta -\mu_{\eta }\right)}^{{\;}^{\prime }}\Sigma_{\eta }^{-1}\left(\eta -\mu_{\eta }\right), \end{array}$$

where ***μ_η_*** and **Σ*****_η_*** are the prior mean vector and the covariance matrix of ***η***, respectively.

### 4.4. SAEM algorithm for estimating Ω and *η*

The SAEM algorithm was first proposed by Delyon et al. (1999), and it replaces the integral calculation with a stochastic approximation in the E-step, which significantly improves computational efficiency, especially for exponential family distributions. We assume that: the iteration has updated to step *k*; **Ω**^(*k*)^ = (***a***^(*k*)^, ***b***^(*k*)^, ***c***^(*k*)^) and ***η***^(*k*)^ are the current estimates of the item parameters and the shape parameter of the Ramsay curve, respectively; and **Ψ**^(*k*)^ = (**Θ**^(*k*)^, **w**^(*k*)^, **z**^(*k*)^) is the current augmented data. The detailed estimation steps of the SAEM algorithm incorporating the Ramsay curve (the RC-SAEM algorithm) at step *k* + 1 are given as follows.

**Simulation step (S-step). Sample Ψ** ^**(** ***k*** **+1)**^ **:**

**S1-step:** Sample $\theta_{i}^{\left(k+1\right)}$

(31) $$\begin{array}{l} P\left(\theta_{i}^{\left(k+1\right)}=x_{q}|{\text{U}}_{i},\Omega^{\left(k\right)},\eta^{\left(k\right)}\right)\\ =\frac{\prod_{j=1}^{J}\left\{P_{j}{\left(x_{q}|\Omega^{\left(k\right)}\right)}^{u_{ij}}{\left[1-P_{j}\left(x_{q}|\Omega^{\left(k\right)}\right)\right]}^{1-u_{ij}}g\left(x_{q}|\eta^{\left(k\right)}\right)\right\}}{\sum_{q=1}^{Q}\prod_{j=1}^{J}\left\{P_{j}{\left(x_{q}|\Omega^{\left(k\right)}\right)}^{u_{ij}}{\left[1-P_{j}\left(x_{q}|\Omega^{\left(k\right)}\right)\right]}^{1-u_{ij}}g\left(x_{q}|\eta^{\left(k\right)}\right)\right\}}, \end{array}$$

**S2-step:** Sample $w_{ij}^{\left(k+1\right)}$

(32) $$\begin{array}{l} w_{ij}^{\left(k+1\right)}|\Omega^{\left(k\right)},\text{U},\Theta^{\left(k+1\right)}\sim \\ \{\begin{array}{ll} \text{Bernoulli}\left(\frac{\Phi \left(a_{j}^{\left(k\right)}\theta_{i}^{\left(k+1\right)}+b_{j}^{\left(k\right)}\right)}{c_{j}^{\left(k\right)}+\left(1-c_{j}^{\left(k\right)}\right)\Phi \left(a_{j}^{\left(k\right)}\theta_{i}^{\left(k+1\right)}+b_{j}^{\left(k\right)}\right)}\right), & u_{ij}=1\\ \text{Bernoulli}\left(0\right). & u_{ij}=0 \end{array} \end{array}$$

**S3-step:** Sample $z_{ij}^{\left(k+1\right)}$

(33) $$\begin{array}{l} \;z_{ij}^{\left(k+1\right)}|\Omega^{\left(k\right)},\Theta^{\left(k\right)},w^{\left(k+1\right)}\sim \\ N(a_{j}^{k}\theta_{i}^{\left(k+1\right)}+b_{j}^{\left(k\right)},1)[I(z_{ij}^{\left(k+1\right)}>0)w_{ij}^{\left(k+1\right)}\\ +I(z_{ij}^{\left(k+1\right)}\leq 0)(1-w_{ij}^{\left(k+1\right)})], \end{array}$$

**Stochastic approximation step (SA-step). Update sufficient statistics** ${\text{S}}_{j}^{(k+1)}$ **(*j* = 1, ⋯ , *J*):**

Based on the factorization theorem, the sufficient statistics of the item parameters **Ω** are

(34) $$\begin{array}{r} S_{j}\left({\text{U},\Psi }^{\left(k+1\right)}\right)=\left(S_{j1}^{\left(k+1\right)},S_{j2}^{\left(k+1\right)},S_{j3}^{\left(k+1\right)},S_{j4}^{\left(k+1\right)}\right)\\ \;\left(j=1,2,\cdots \;,J\right), \end{array}$$

and

(35) $$\begin{array}{l} S_{j1}^{\left(k+1\right)}={\text{S}}^{{*}^{\prime }\left(k+1\right)}{\text{S}}^{*\left(k+1\right)}, \end{array}$$

(36) $$\begin{array}{l} S_{j2}^{\left(k+1\right)}={\text{S}}^{{*}^{\prime }\left(k+1\right)}{\text{z}}_{j}^{\left(k+1\right)}, \end{array}$$

(37) $$\begin{array}{l} S_{j3}^{\left(k+1\right)}=\overset{N}{\underset{i=1}{\sum }}\left(1-w_{ij}^{\left(k+1\right)}\right), \end{array}$$

(38) $$\begin{array}{l} S_{j4}^{\left(k+1\right)}=\overset{N}{\underset{i=1}{\sum }}\left(1-w_{ij}^{\left(k+1\right)}\right)u_{ij}, \end{array}$$

where S^*(*k*+1)^ = (**Θ**^(*k*+1)^,**1**_*N*_), **1**_*N*_ is a unit column vector with dimension *N*, and the vector ${\text{z}}_{j}^{\left(k+1\right)}$ is the *j*th column of augmented data set **z**^(*k*+1)^ in the *k* + 1th iteration.

Thus, the stochastic approximation step is:

(39) $$\begin{array}{l} S_{j}^{\left(k+1\right)}=S_{j}^{\left(k\right)}+\gamma_{k}\left[S_{j}\left(\text{U},\Psi^{\left(k+1\right)}\right)-S_{j}^{\left(k\right)}\right], \end{array}$$

where **Ψ**^(*k*+1)^ = (**Θ**^(*k*+1)^, **w**^(*k*+1)^, **z**^(*k*+1)^) is the augmented data sets that are simulated from the S-step, and {γ_*k*_, *k* = 1, 2, ⋯ } is a decreasing sequence of gain constants, as defined by Robbins and Monro (1951), which satisfies

$$\begin{array}{l} \gamma_{k}\epsilon \left(0,1\right],\overset{\infty }{\underset{k=1}{\sum }}\gamma_{k}=\infty ,\overset{\infty }{\underset{k=1}{\sum }}\gamma_{k}^{2}<\infty . \end{array}$$

**Maximization step (M-step). Update Ω**^**(*****k*****+1)**^ **and** ***η***^**(*****k*****+1)**^: **M1-step**. Update **Ω**^**(*****k*****+1)**^

Based on *L*(**Ω**|**U**, **Ψ**) and the prior distributions in Equations (10) and (11), the posterior distributions of **Ω**_***j***_ = (*a*_*j*_, *b*_*j*_, *c*_*j*_) are

(40) $$\begin{array}{l} {\left(a_{j},b_{j}\right)}^{\prime }|{\text{U},\Psi }^{\left(k+1\right)}\sim N_{2}\left(\mu^{*\left(k+1\right)},\Sigma^{*\left(k+1\right)}\right)I\left(a_{j}>0\right) \end{array}$$

and

(41) $$\begin{array}{l} c_{j}|{\text{U},\Psi }^{\left(k+1\right)}\sim \text{Beta}\left(\alpha^{*\left(k+1\right)},\beta^{*\left(k+1\right)}\right), \end{array}$$

where

$$\begin{array}{c} \mu^{*\left(k+1\right)}={\left(S_{j1}^{\left(k+1\right)}+\Sigma \right)}^{-1}\left(S_{j2}^{\left(k+1\right)}+\Sigma^{-1}\mu \right),\\ \Sigma^{*\left(k+1\right)}={\left(S_{j1}^{\left(k+1\right)}+\Sigma^{-1}\right)}^{-1},\\ \alpha^{*\left(k+1\right)}=\alpha +S_{j4}^{\left(k+1\right)},\\ \beta^{*\left(k+1\right)}=\beta +S_{j3}^{\left(k+1\right)}-S_{j4}^{\left(k+1\right)}. \end{array}$$

***μ***, **Σ**, α, β are the hyper-parameters in prior distributions of ***a, b, c***, please refer to Equations (10) and (11).

Thus, the MAP estimates of *a*_*j*_, *b*_*j*_, and *c*_*j*_ for the *k* + 1th iteration are

(42) $$\begin{array}{l} {â}_{j}^{\left(k+1\right)}=\mu^{*\left(k+1\right)}\left[1\right]I\left(\mu^{*\left(k+1\right)}\left[1\right]>0\right)+\delta \\ \times I\left(\mu^{*\left(k+1\right)}\left[1\right]\leq 0\right), \end{array}$$

(43) $$\begin{array}{l} {\hat{b}}_{j}^{\left(k+1\right)}=\mu^{*\left(k+1\right)}\left[2\right], \end{array}$$

(44) $$\begin{array}{l} {ĉ}_{j}^{\left(k+1\right)}=\frac{\alpha^{*\left(k+1\right)}+S_{j4}^{\left(k+1\right)}-1}{\alpha^{*\left(k+1\right)}+\beta^{*\left(k+1\right)}+S_{j3}^{\left(k+1\right)}-2}, \end{array}$$

where δ is a tiny positive number to satisfy *a*_*j*_ > 0, μ^*(*k*+1)^[1] denotes the first element of μ^*(*k*+1)^ in the *k* + 1th iteration, and μ^*(*k*+1)^[2] denotes the second element of μ^*(*k*+1)^ in the *k* + 1th iteration.

**M2-step**. Update ***η***^(*k*+1)^:

Update ***η***^(*k*+1)^ according to the Newton–Raphson iteration, which satisfies

(45) $$\begin{array}{l} \eta^{\left(t+1\right)}=\eta^{\left(t\right)}-{\left(\frac{\partial^{2}l_{R}}{\partial \eta \partial \eta^{{\;}^{\prime }}}\right)}^{-1}\frac{\partial l_{R}}{\partial \eta }, \end{array}$$

where *l*_*R*_ is the logarithm RCP of ***η*** in Equation (30). Note that *t* is the number of iterations in the process of implementing the Newton-Raphson iteration algorithm. Let ***η***^(*k*+1)^ = ***η***^(*t*+1)^ when Newton-Raphson iteration algorithm reaches convergence after executing *t* + 1 iterations of inner loop and continue the computations of SAEM algorithm.

Repeat the S-step, SA-step, and M-step until the convergence criteria are satisfied. Here, the SAEM algorithm is considered to have converged when the maximum absolute difference of the MAP estimates between two adjacent iterations (i.e., max ∣***ζ***^(*k*)^ − ***ζ***^(*k*+1)^∣) is less than 10^−4^ or the maximum number of iterations (selected as 2500) is reached.

Note that the augmented data sets in the S-step can be simulated *m*_*k*_ sets in the original SAEM algorithm (Delyon et al., 1999), that is, $\Psi_{p}^{\left(k+1\right)}=\left(\Theta_{p}^{\left(k+1\right)},{\text{w}}_{p}^{\left(k+1\right)},{\text{z}}_{p}^{\left(k+1\right)}\right)$ (*p* = 1, 2, ⋯ , *m*_*k*_). In this case, $S_{j}\left(\text{U},{\text{Ψ}}^{\left(k+1\right)}\right)$ in Equation (39) can be replaced by the average value of the *m*_*k*_ updated sufficient statistics computed from these augmented data sets, that is, $\frac{\overset{m_{k}}{\underset{p=1}{\sum }}S_{j}\left(\text{U},\Psi_{p}^{\left(k+1\right)}\right)}{m_{k}}$. According to previous studies of the SAEM algorithm, the number of simulations *m*_*k*_ = 1 is suggested to be set for all the iterations (Delyon et al., 1999; Kuhn and Lavielle, 2004), which makes the M-step straightforward to implement and increases the computational efficiency. In most cases, the increasing of *m*_*k*_ will not improve the accuracy of the algorithm. For the Robbins–Monro gain coefficient, let $\gamma_{k}={\left(\frac{1}{k}\right)}^{\alpha }$, where α ⩾ 0. A larger step size (that is, α = 0) can accelerate the rate of convergence, but this will result in inflation of the Monte Carlo error introduced when approximating the integral by the average of a set of simulations in the SA-step (Jank, 2006). A smaller step size (that is, α = 1) may allow the sequence of estimates to approach the neighborhood of the solution with a small Monte Carlo error, but it will also slow down the convergence rate (Jank, 2006; Geis, 2019). In this work, the step size γ_*k*_ was chosen to be 1 in the first 1,000 iterations to ensure that enough steps were used when quickly approaching the neighborhood of the solution, but this also inflates the Monte Carlo error at the same time (Jank, 2006). Then, we let $\gamma_{k}=\frac{1}{k-1000}$ when *k* > 1,000 to rapidly reduce the Monte Carlo error of the estimates, though this slows down the convergence rate (Gu and Zhu, 2001; Kuhn and Lavielle, 2004; Jank, 2006; Geis, 2019).

### 4.5. Evaluation criteria

In fact, researchers have conducted in-depth studies on model selection methods based on evidence function (log-likelihood function), such as likelihood ratio test (LR-test) and chi-square difference test. However, these methods actually have some drawbacks and limitations. As depicted in Woods (2006), LR-test is not an ideal evaluation criterion for RC-IRT for two reasons. One is its tendency to select large models. It tends to select the largest model that is significantly better than the true model. Another limitation is that, like all chi-square difference tests, it requires the larger model to fit the data in an absolute sense, which is difficult to establish. A chi-square test of absolute fit is usually not appropriate in IRT because the number of possible response patterns is large, and the probability of any one of the patterns is small; thus, statistics like Pearson's are not chi-square distributed (Maydeu-Olivares and Cai, 2006). For these reasons, LR tests alone should not be relied upon for model selection. Thus, Woods (2006) considered the following three model selection criteria.

Three model selection criteria—Akaike's information criterion (AIC; Akaike, 1973), Bayesian information criterion (BIC; Schwarz, 1978), and Hannan–Quinn information criterion (HQIC; Hannan, 1987; Woods, 2007, 2008)—are considered:

(46) $$\begin{array}{l} \text{AIC}=-2\log L+\log\left(N\right), \end{array}$$

(47) $$\begin{array}{l} \text{BIC}=-2\log L+n\log\left(N\right), \end{array}$$

(48) $$\begin{array}{l} \text{HQIC}=-2\log L+2n\log\left(\log\left(N\right)\right), \end{array}$$

where log *L* is the log-likelihood of all the parameters **Ω** and ***η***, *n* is the number of parameters, and *N* is the sample size.

As the number of *konts* and *degree* increases, the number of free parameters increases and the goodness of fitting is improved. AIC (BIC and HQIC) encourages the goodness of data fitting (information provided by the evidence function) but tries to avoid overfitting (prevent the cases of too many free parameters). The purpose of information criterion is to find the balance between model fit and model complexity. The preferred model should be the one with the lowest AIC (BIC and HQIC) values.

To evaluate the accuracy of the item parameter recoveries, the bias and root-mean-square error (RMSE) are calculated. Supposing *R* is the number of replications, the bias of parameter ω is

(49) $$\begin{array}{l} \text{Bias}=\frac{1}{R}\overset{R}{\underset{r=1}{\sum }}\left({\hat{\omega }}_{r}-\omega \right), \end{array}$$

and the RMSE of ω is defined as

(50) $$\begin{array}{l} \text{RMSE}=\sqrt{\frac{1}{R}\overset{R}{\underset{r=1}{\sum }}{\left({\hat{\omega }}_{r}-\omega \right)}^{2}}, \end{array}$$

where $\hat{\omega_{r}}$ is the parameter estimate at the r*th* replication and ω is the true value of the parameter.

Since the scales of the true RC parameters are complicated and difficult to handle, the bias and RMSE are less appropriate measures of recovery accuracy. Instead, the integrated square error (ISE),

(51) $$\begin{array}{l} \text{ISE}\left(ĝ\right)=\int {\left[g\left(\theta |\hat{\eta }\right)-g\left(\theta |\eta \right)\right]}^{2}d\theta , \end{array}$$

is used to measure the discrepancy between the true and estimated RCs, as used by Woods and Lin (2009) and Monroe and Cai (2014). The ISE was multiplied by 1,000 to facilitate comparison. The values of ISE were computed across all replications.

## 5. Simulation studies

### 5.1. Simulation study 1

The first simulation study was performed to select the optimal numbers of *knots* and the *degree* of the B-spline functions for the RC-3PNO model based on three model selection criteria—the AIC, BIC, and HQIC—as well as to show the item parameter recoveries when the true ability density is normal, skewed, or bimodal.

#### 5.1.1. Design

The true latent-ability densities were represented by rectangular quadrature points, ranging from −6 to 6 in steps of 0.1. For the skewed and bimodal cases, the true ability density was generated by mixing two normal densities, that is, $p_{1}N\left(\mu_{1},\sigma_{1}^{2}\right)+p_{2}N\left(\mu_{2},\sigma_{2}^{2}\right)$, in which *p*_1_ + *p*_2_ = 1. For the skewed density, the generating parameters were: μ_1_ = −2.7, ${\sigma_{1}}^{2}=0.2$, μ_2_ = 1.1, and ${\sigma_{2}}^{2}=1.1$. The skewness and kurtosis of θ were 2.46 and 8.45, respectively. For the bimodal density, the generating parameters were: μ_1_ = −2, ${\sigma_{1}}^{2}=0.25$, μ_2_ = 2.5, and ${\sigma_{2}}^{2}=0.5$. In this case, the skewness and kurtosis of θ were 1.45 and 4.21, respectively. The true item parameters were set to be consistent with common practice in IRT. The discrimination parameters *a* were sampled from *U*(1, 2.5), the intercept parameters *b* were simulated from *N*(0, 1), and the guessing parameters *c* were generated from Beta(5, 17).

For each of the three true latent-ability densities, 10 models with different combinations of *knots* and *degree* were fitted to the generated data. The *degree* values of the B-spline functions were either 3 or 4, and the number of *knots* was chosen to be between 2 and 6; these are the typical choices when estimating ***η*** in RC-IRT (Woods and Thissen, 2006; Monroe and Cai, 2014). The sample size was set to be 1,000, and the test length was fixed at 30. Therefore, there were 30 simulation conditions, and each simulation condition was conducted 100 times.

#### 5.1.2. Results

Table 1 presents the model selection results when the true latent-ability densities are normal, skewed, and bimodal. The bold-faced values indicate the smallest AIC, BIC, and HQIC values in each column. In addition, under each of the three shapes of ability density, the AIC, BIC, and HQIC are consistent to choose one common combination of the *knots* and *degree*.

**Table 1:** Model selection results under different combinations of *knots* and *degree* when θ is normal, skewed, or bimodal.

	**Normal**				**Skewed**				**Bimodal**		
	**AIC**	**BIC**	**HQIC**		**AIC**	**BIC**	**HQIC**		**AIC**	**BIC**	**HQIC**
2knots-degree3	34121.1	34582.5	34296.5		33954.3	34415.6	34129.6		32576.4	33037.7	32751.7
2knots-degree4	34099.8	34566.1	34277.0		33968.3	34434.5	34145.5		32639.4	33105.6	32816.6
3knots-degree3	34061.1	34531.7	34238.3		33815.8	34282.0	33993.0		32531.3	32997.6	32708.5
3knots-degree4	34089.8	34561.0	34268.9		33913.9	34385.0	34092.9		32499.8	32970.9	32678.8
4knots-degree3	34117.4	34588.5	34296.4		33762.6	34233.7	33941.7		32381.9	32853.0	32561.0
4knots-degree4	**34055.6**	**34527.4**	**34236.5**		33745.7	34221.8	33926.7		32456.2	32932.3	32637.2
5knots-degree3	34080.2	34556.3	34261.1		33715.4	34191.8	33896.4		**32214.9**	**32690.9**	**32395.8**
5knots-degree4	34119.7	34600.6	34302.5		33786.8	34267.8	33969.6		32268.4	32749.3	32451.1
6knots-degree3	34113.2	34594.1	34296.0		**33710.9**	**34191.5**	**33893.7**		32241.5	32722.5	32424.3
6knots-degree4	34068.5	34554.4	34253.2		33741.0	34226.9	33925.7		32317.9	32803.7	32502.5

The boldfaced values indicate the smallest AIC, BIC, and HQIC values in each column.

When the true ability density is normal, all three model selection criteria result in the RC-3PNO model with 4 *knots* and a *degree* of 4 (denoted as the 4-4 RC-3PNO model) being chosen as the best-fitting model. However, the differences in the model selection results under each condition are slight overall. When the true ability density is skewed, the values of AIC, BIC, and HQIC are the smallest for the RC-3PNO model with 6 *knots* and a *degree* of 3 (denoted as the 6-3 RC-3PNO model); the best model chosen in the bimodal case is the RC-3PNO model with 5 *knots* and a *degree* of 3 (denoted as the 5-3 RC-3PNO model). In addition, in the skewed and bimodal cases, the values of AIC, BIC, and HQIC have obvious discrepancies across different combinations of *knots* and *degrees* for the RC-3PNO model. Specifically, for the skewed case, the model selection results for the RC-3PNO model with *knots* and *degree* combinations 2-3, 2-4, and 3-4 show relatively large values compared with the best-fitting model, i.e., the 6-3 RC-3PNO model, with discrepancies over 200. The reason for this may be that a *knots* value of 2 or 3 is not sufficient to describe a skewed ability density. The RC-3PNO models with *knots* and *degree* combinations of 4-3, 4-4, 5-3, 5-4, 6-3, and 6-4 result in very little differences in the AIC, BIC, and HQIC values when the true ability density is skewed. For the bimodal case, the discrepancies in model selection results between the RC-3PNO models with *knots* and *degree* combinations of 2-3, 2-4, 3-3, 3-4, and 4-4 and the best-fitting model, i.e., the 5-3 RC-3PNO model, are greater than 200. The differences in model selection results between the models with the combinations 5-4 and 6-3 and the best-fitting model (5-3) are extremely small.

**Figure 1:**
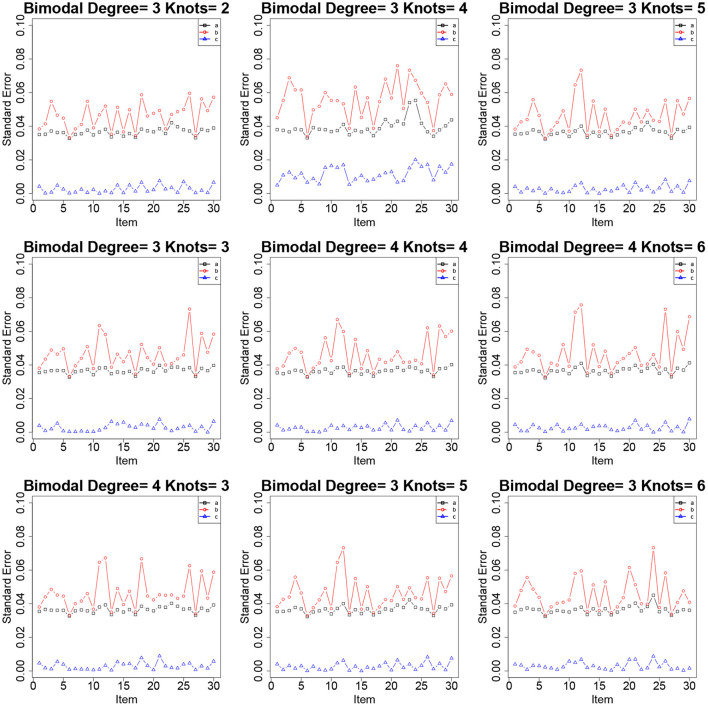
The standard errors of item parameters using the RC-SAEM algorithm when the latent trait is bimodal in simulation study 1.

**Figure 2:**
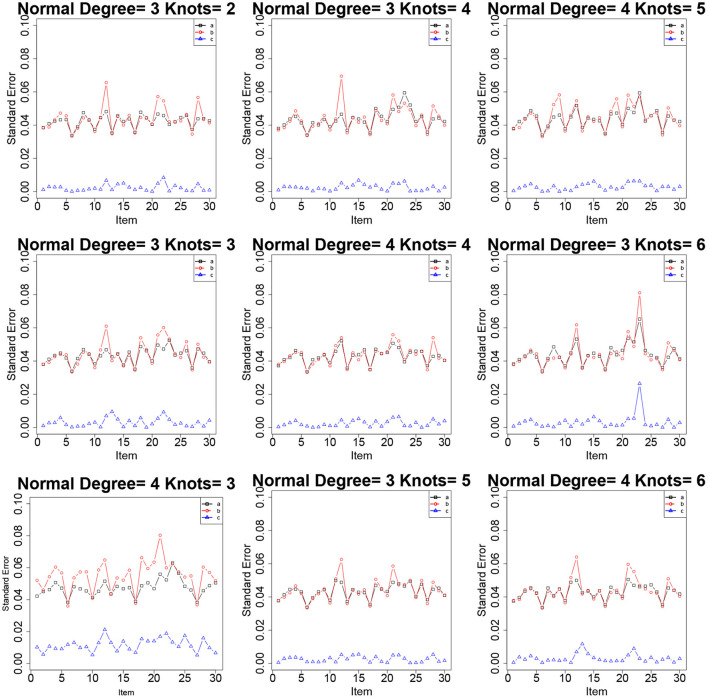
The standard errors of item parameters using the RC-SAEM algorithm when the latent trait is normal in simulation study 1.

**Figure 3:**
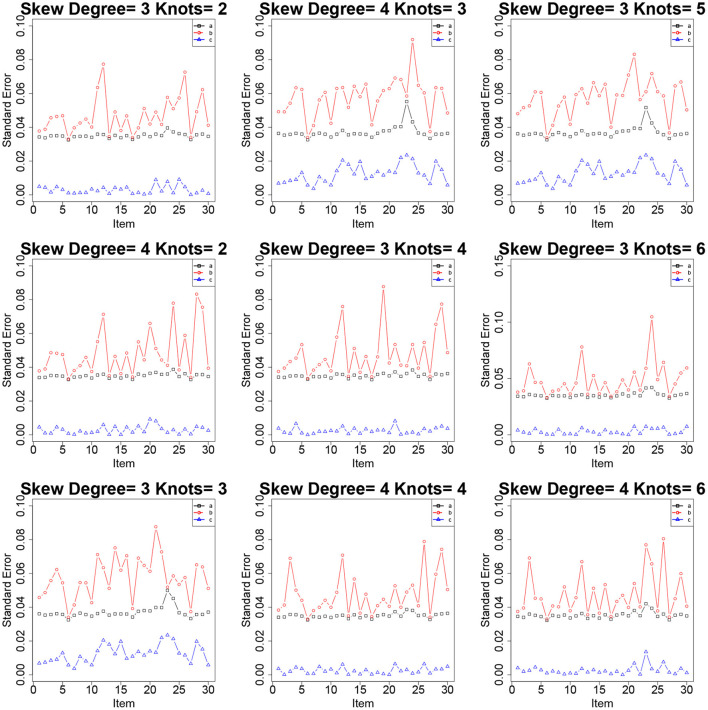
The standard errors of item parameters using the RC-SAEM algorithm when the latent trait is skew in simulation study 1.

Tables 2–4 show the item parameter estimation results. There are no distinct differences in the bias and RMSE values of item parameters *a*, *b*, and *c* for the fitted models across all the conditions; however, some subtle variations still exist. Thus, the choices of *knots* and *degree* in this simulation study had no noticeable influences on the bias and RMSE values of the item parameters. The standard errors of item parameters are presented in Figures 1–3. In the majority of cases, the standard errors of item parameters *a* and *b* are below 0.08, and the standard errors of parameter *c* are below 0.02, which are within the tolerable ranges. It indicates that the RC-SAEM algorithm performs well in estimation stability.

**Table 2:** Bias and RMSE of item parameter estimates under different combinations of knots and degree in normal case.

	***a***			***b***			***c***	
	**Bias**	**RMSE**		**Bias**	**RMSE**		**Bias**	**RMSE**
2knots-degree3	0.101	0.354		−0.023	0.366		−0.006	0.069
2knots-degree4	0.091	0.329		−0.004	0.357		−0.009	0.070
3knots-degree3	0.086	0.299		−0.006	0.333		−0.008	0.068
3knots-degree4	0.084	0.325		−0.012	0.342		−0.007	0.071
4knots-degree3	0.081	0.310		−0.025	0.346		−0.025	0.068
4knots-degree4	0.079	0.308		0.005	0.334		−0.011	0.067
5knots-degree3	0.094	0.349		−0.032	0.367		−0.009	0.067
5knots-degree4	0.081	0.321		−0.026	0.354		−0.008	0.068
6knots-degree3	0.083	0.299		−0.016	0.335		−0.009	0.067
6knots-degree4	0.078	0.298		−0.014	0.337		−0.008	0.068

**Table 3:** Bias and RMSE of item parameter estimates under different combinations of knots and degree in skewed case.

	***a***			***b***			***c***	
	**Bias**	**RMSE**		**Bias**	**RMSE**		**Bias**	**RMSE**
2knots-degree3	0.046	0.276		−0.065	0.352		−0.007	0.080
2knots-degree4	0.039	0.261		−0.004	0.330		−0.015	0.078
3knots-degree3	0.041	0.251		0.034	0.329		−0.018	0.082
3knots-degree4	0.042	0.268		−0.046	0.339		−0.008	0.079
4knots-degree3	0.047	0.262		−0.028	0.363		−0.008	0.082
4knots-degree4	0.056	0.282		−0.024	0.370		−0.008	0.083
5knots-degree3	0.043	0.266		−0.017	0.381		−0.010	0.082
5knots-degree4	0.052	0.280		−0.042	0.367		−0.007	0.082
6knots-degree3	0.055	0.285		−0.043	0.377		−0.007	0.080
6knots-degree4	0.051	0.276		−0.060	0.393		−0.005	0.084

**Table 4:** Bias and RMSE of item parameter estimates under different combinations of knots and degree in bimodal case.

	***a***			***b***			***c***	
	**Bias**	**RMSE**		**Bias**	**RMSE**		**Bias**	**RMSE**
2knots-degree3	0.078	0.304		−0.039	0.369		−0.011	0.080
2knots-degree4	0.084	0.311		−0.016	0.367		−0.015	0.081
3knots-degree3	0.095	0.324		0.048	0.363		−0.020	0.084
3knots-degree4	0.075	0.299		−0.016	0.374		−0.012	0.079
4knots-degree3	0.087	0.324		−0.039	0.429		−0.008	0.087
4knots-degree4	0.087	0.319		0.038	0.367		−0.017	0.084
5knots-degree3	0.071	0.303		−0.029	0.420		−0.011	0.087
5knots-degree4	0.076	0.304		−0.024	0.423		−0.008	0.086
6knots-degree3	0.073	0.310		−0.047	0.489		−0.010	0.087
6knots-degree4	0.082	0.310		−0.012	0.410		−0.013	0.084

### 5.2. Simulation study 2

This simulation study was conducted to compare the performance of the proposed RC-SAEM algorithm, the original SAEM, and the MCMC algorithm in estimating the item parameters of the 3PNO model.

#### 5.2.1. Design

The shapes of the true ability density, *g*(θ), were set to be normal, skewed, and bimodal distributions (Woods and Thissen, 2006; Monroe and Cai, 2014). For the skewed case, the skewness and kurtosis of θ were 1.72 and 9.16, respectively; in the bimodal case, the skewness and kurtosis of θ were 0.95 and 2.74, respectively. The numbers of examinees were set to be 500, 1,000, and 2,000 to represent small, medium, and large sample sizes, respectively. The test lengths were set as 15 and 30. Therefore, a total of 18 simulation conditions were manipulated. Each simulation condition was replicated 100 times.

The data-generating model and the fitted model were the same, that is, the 6-3 RC-3PNO model (6 *knots* with a *degree* of 3). As noted in the description of the model, 121 discrete points [i.e., *x*_*q*_ (*q* = 1, 2, ⋯ , *Q*), and *Q* = 121] from −6 to 6 in steps of 0.1 were used to describe the true ability density. After the true value of ***η*** was selected, the true ability of θ_*i*_ was manipulated according to grid sampling, similar to the S1 step of the RC-SAEM algorithm. That is, first, a true ***η*** value was chosen corresponding to the given shape of *g*(*x*_*q*_|***η***). Then, the probabilities of θ_*i*_ = *x*_*q*_ in the grid sampling were set to *g*(*x*_*q*_|***η***) (*q* = 1, 2, ⋯ , *Q*). The values of θ_*i*_ (*i* = 1, 2, ⋯ , *n*) were standardized to have a mean of 0 and a standard deviation of 1. The values after standardization were chosen to be true values of Θ. The true item parameters were the same as those in simulation study 1.

To avoid the effects of the choice of prior distribution on the estimation results, the priors for *a* and *b* were chosen to be non-informative priors. The prior for the *c* parameter was set to be Beta(5, 17) (the mean is $\frac{5}{5+17}=0.227$), which is consistent with the common prior choice in IRT (Harwell and Baker, 1991; Béguin and Glas, 2001) because the nominal guessing probability is around 0.25 for multiple-choice items with four options. These priors for the item parameters were adopted in the SAEM, RC-SAEM, and MCMC algorithms.

#### 5.2.2. Results

Tables 5, 6 show the bias and RMSE values of the item parameter estimates under different simulation conditions. For simplicity, the average values of the bias and RMSE across *J* items are presented. For the normal distribution of the ability density, the bias and RMSE values of parameters *a* and *b* from the RC-SAEM algorithm are smaller than those from the SAEM algorithm. The bias values of the *a* parameter from the MCMC algorithm are larger than those from the other two algorithms under all of the three sample sizes. We can see that the estimation results of the MCMC algorithm are not very satisfactory when the sample size is 500 and the test length is 15; this indicates that a sample size of 500 is not large enough for precise estimation of the 3PNO model using MCMC with non-informative priors on *a* and *b*. The poor performance of the MCMC algorithm for the normal distribution case may be due to the choice of non-informative priors on the parameters *a* and *b*. Therefore, the RC-SAEM algorithm performs best when the true θ density is normal. In addition, the RMSEs of parameters *a* and *b* show an approximately decreasing trend as the sample size increases.

**Table 5:** Bias and RMSE of the item parameter estimates and the values of ISE statistic under different ability densities when the test length is 30.

			***a***			***b***			***c***		**ISE**
			**Bias**	**RMSE**		**Bias**	**RMSE**		**Bias**	**RMSE**	
*N* = 500	Normal	SAEM	0.044	0.322		-0.059	0.305		-0.010	0.069	-
		RC-SAEM	0.031	0.261		−0.008	0.236		−0.014	0.067	0.061
		MCMC	0.051	0.265		−0.082	0.294		0.012	0.057	-
	Skewed	SAEM	0.096	0.508		-0.387	0.584		0.016	0.084	-
		RC-SAEM	0.030	0.261		−0.021	0.239		−0.017	0.071	−0.006
		MCMC	0.112	0.433		−0.399	0.592		0.034	0.071	-
	Bimodal	SAEM	0.094	0.350		-0.059	0.405		−0.012	0.079	−
		RC-SAEM	0.097	0.376		−0.024	0.471		-0.012	0.083	−0.143
		MCMC	0.154	0.370		-0.191	0.383		0.038	0.070	−
*N* = 1, 000	Normal	SAEM	0.017	0.202		−0.044	0.218		−0.011	0.060	-
		RC-SAEM	0.015	0.186		0.001	0.185		−0.015	0.059	0.027
		MCMC	0.023	0.170		-0.002	0.174		0.003	0.047	-
	Skewed	SAEM	0.043	0.334		−0.323	0.436		0.022	0.083	−
		RC-SAEM	0.014	0.190		0.001	0.187		−0.018	0.072	−0.039
		MCMC	0.056	0.286		−0.313	0.415		0.034	0.065	-
	Bimodal	SAEM	0.067	0.275		−0.072	0.342		-0.005	0.072	-
		RC-SAEM	0.074	0.281		−0.025	0.385		−0.010	0.075	−0.035
		MCMC	0.157	0.316		-0.240	0.370		0.047	0.072	−
*N* = 2, 000	Normal	SAEM	0.012	0.152		−0.018	0.155		−0.012	0.057	-
		RC-SAEM	0.009	0.136		0.012	0.139		-0.014	0.055	0.008
		MCMC	0.015	0.137		−0.038	0.154		0.004	0.041	-
	Skewed	SAEM	0.024	0.256		-0.294	0.362		0.032	0.078	-
		RC-SAEM	0.009	0.148		0.000	0.154		−0.015	0.066	−0.007
		MCMC	0.026	0.203		-0.261	0.318		0.039	0.059	-
	Bimodal	SAEM	0.060	0.249		−0.075	0.314		−0.002	0.065	-
		RC-SAEM	0.056	0.237		0.001	0.324		-0.006	0.073	0.087
		MCMC	0.223	0.343		−0.315	0.460		0.067	0.080	-

*N* denotes the sample size, normal, skewed, and bimodal refers to the shape of the ability density. The SAEM refers to estimating 3PNO model using SAEM, RC-SAEM refers to estimating RC-3PNO model using SAEM, and the MCMC refers to estimating 3PNO model using MCMC algorithm.

**Table 6:** Bias and RMSE of the item parameter estimates and the values of ISE statistic under different ability densities when the test length is 15.

			***a***			***b***			***c***		**ISE**
			**Bias**	**RMSE**		**Bias**	**RMSE**		**Bias**	**RMSE**	
*N* = 500	Normal	SAEM	0.111	0.586		−0.106	0.362		−0.007	0.074	−
		RC-SAEM	0.052	0.385		-0.054	0.286		−0.008	0.068	0.061
		MCMC	0.153	0.439		−0.110	0.414		0.011	0.066	-
	Skewed	SAEM	0.134	0.740		-0.423	0.652		0.017	0.093	−
		RC-SAEM	0.038	0.345		−0.066	0.303		-0.006	0.071	-0.006
		MCMC	0.191	0.473		−0.411	0.585		0.028	0.073	-
	Bimodal	SAEM	0.068	0.362		−0.104	0.292		−0.023	0.066	−
		RC-SAEM	0.058	0.363		0.015	0.334		−0.034	0.073	−0.143
		MCMC	0.131	0.350		−0.060	0.326		0.011	0.063	-
*N* = 1, 000	Normal	SAEM	0.036	0.287		-0.053	0.259		−0.008	0.067	−
		RC-SAEM	0.050	0.277		−0.013	0.227		−0.008	0.068	0.027
		MCMC	0.106	0.299		0.011	0.229		0.001	0.056	-
	Skewed	SAEM	0.057	0.453		−0.366	0.502		0.014	0.083	−
		RC-SAEM	0.020	0.247		−0.037	0.239		−0.011	0.072	−0.011
		MCMC	0.134	0.327		−0.308	0.432		0.026	0.065	−
	Bimodal	SAEM	0.053	0.289		−0.128	0.255		−0.012	0.059	-
		RC-SAEM	0.049	0.332		0.010	0.276		−0.032	0.076	-0.035
		MCMC	0.107	0.284		−0.058	0.292		0.014	0.059	−
*N* = 2, 000	Normal	SAEM	0.034	0.244		−0.027	0.207		−0.009	0.065	−
		RC-SAEM	0.029	0.191		0.021	0.176		-0.009	0.062	0.008
		MCMC	0.088	0.210		−0.043	0.179		0.003	0.049	-
	Skewed	SAEM	0.025	0.360		−0.306	0.400		0.025	0.082	-
		RC-SAEM	0.010	0.188		−0.004	0.187		−0.013	0.071	−0.007
		MCMC	0.108	0.272		−0.257	0.363		0.032	0.061	-
	Bimodal	SAEM	0.048	0.269		-0.161	0.249		-0.007	0.062	-
		RC-SAEM	0.038	0.284		0.017	0.226		−0.030	0.075	0.087
		MCMC	0.095	0.275		−0.081	0.314		0.021	0.061	−

*N* denotes the sample size, normal, skewed, and bimodal refers to the shape of the ability density. The SAEM refers to estimating 3PNO model using SAEM, RC-SAEM refers to estimating RC-3PNO model using SAEM, and the MCMC refers to estimating 3PNO model using MCMC algorithm.

In the cases of the skewed and bimodal distributions, the bias and RMSE values of *a* and *b* from the RC-SAEM algorithm are noticeably lower than those from the SAEM and MCMC algorithms, indicating that the proposed RC-SAEM algorithm is effective for skewed and bimodal densities. It is worth noting that the bias values of *a* from the MCMC algorithm are larger than those from the RC-SAEM and SAEM algorithms, while the corresponding RMSEs are smaller than those from the SAEM algorithm in a few conditions, which demonstrates that the estimation of *a* parameter under the MCMC algorithm in skewed and bimodal cases is less accurate than the other two algorithms. Although the RMSEs of *a* parameter from the MCMC algorithm are slightly smaller than those from the RC-SAEM algorithm in bimodal cases when the test length is 15 in Table 6, the bias of *a* under RC-SAEM still has an obvious advantage over that of MCMC.

The advantage of RC-SAEM is most evident when considering the bias and RMSE values of parameter *b* when the true ability density is skewed or bimodal. Specifically, for the skewed ability density, the bias and RMSE values of the *b* parameter from the RC-SAEM algorithm are markedly less than those from the SAEM and MCMC algorithms. In addition, for the bimodal latent-ability density, although the RMSE of *b* has no marked differences from the SAEM and RC-SAEM algorithms, the bias of *b* from the RC-SAEM algorithm is still lower than those from the other two. As can be seen from Table 5, in the case of the skewed ability density with a sample size 1,000, the SAEM and MCMC algorithms show obvious biased values on *b* with the absolute values over 0.3; in contrast, the RC-SAEM algorithm shows precise estimates of *b*, with a bias of 0.001 and an RMSE of 0.187. In general, the proposed RC-SAEM algorithm has a distinct advantage over the SAEM and MCMC algorithms in terms of the biases of *a* and *b*. The differences in the bias and RMSE of *c* from the RC-SAEM and SAEM algorithms are very small. The RMSE of *c* from the MCMC algorithm is slightly larger than those from the other two algorithms in the skewed and bimodal cases.

**Figure 4:**
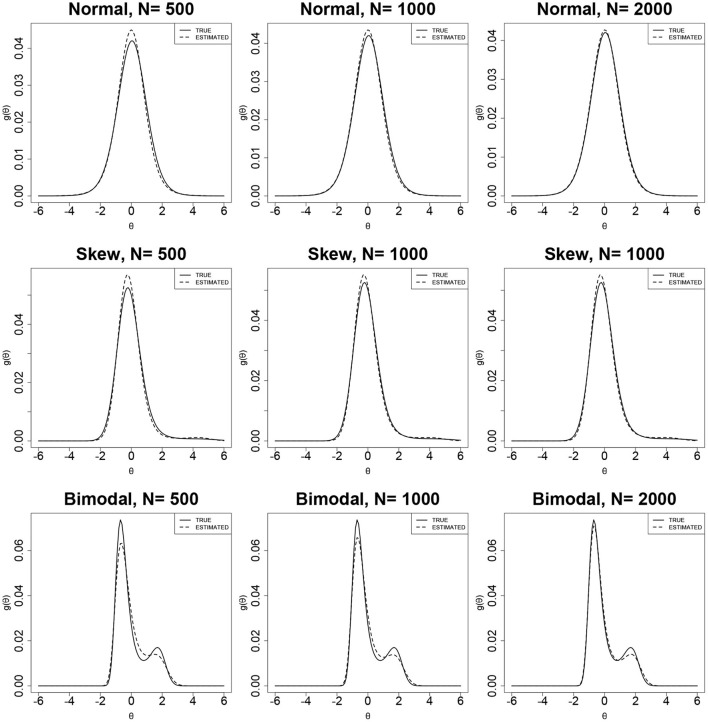
True and estimated density curves using the RC-SAEM algorithm when test length is 30.

**Figure 5:**
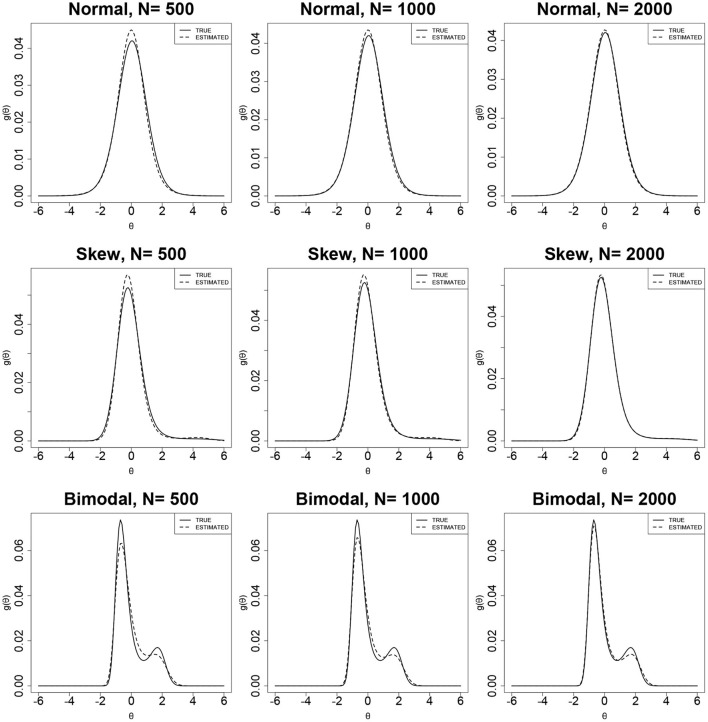
True and estimated density curves using the RC-SAEM algorithm when test length is 15.

Figures 4, 5 show the true and estimated latent-ability density curves when the true ability densities are normal, skewed, or bimodal. The true and estimated latent-ability density curves are almost coincident when the true ability density is skewed, and the two curves show only slight differences when the true latent-ability density is bimodal. In addition, the estimation results of item parameters and the ISE values for 15 items in Table 6 are generally similar to those of 30 items in Table 5. This shows that the accuracy and precision of the RC parameters from the RC-SAEM algorithm are not markedly influenced by the number of items. This result indicates that 15 items is sufficient for the proposed RC-SAEM algorithm to provide satisfactory estimation of the RC parameters for the RC-3PNO model. In contrast to the previous methods in RC-IRT (Woods, 2008), our proposed RC-SAEM algorithm has obvious advantages in terms of estimating RC parameters with relatively short test lengths. For the space limitation, the standard errors of item parameters are presented in Figures A1–A4 of the Appendix. As can be seen from the figures, the standard errors of item parameters show a decreasing trend as the sample size increases. Moreover, the standard errors of the item parameters under all conditions are within reasonable ranges.

## 6. Empirical study

A real data set from the computer-based mathematics assessment of the Programme for International Student Assessment (PISA 2018; OECD, 2019) in China was analyzed. Binary responses from 872 subjects on 11 items in one test form were selected. The SAEM algorithm was fitted to the RC-3PNO and 3PNO models for the real data set. The specifications of the priors of the model parameters were the same as those used in simulation study 2. The same three model selection criteria (AIC, BIC, and HQIC) were used to assess the fit of the model to the real data.

Among the RC-3PNO models with different *knots* and *degree* values (i.e., the 10 combinations used in simulation study 1), that with 2 *knots* and a *degree* of 3 yielded the lowest AIC, BIC, and HQIC values. Thus, this model was selected as the best-fitting model in the subsequent analysis. Table 7 shows the model selection results for this 2-3 RC-3PNO model and the 3PNO model. As shown, the values of all three model selection criteria are smaller for the RC-3PNO model than the 3PNO model. This indicates that the RC-3PNO model gives a better model fit than the 3PNO model for this real data set.

**Table 7:** Model selection results for the real data set.

	**Model parameters**	**−2log *L***	**AIC**	**BIC**	**HQIC**
RC-3PNO	34	9664.3	9738.3	9914.9	9805.9
3PNO	30	10196.1	10262.1	10419.5	10322.3

**Figure 6:**
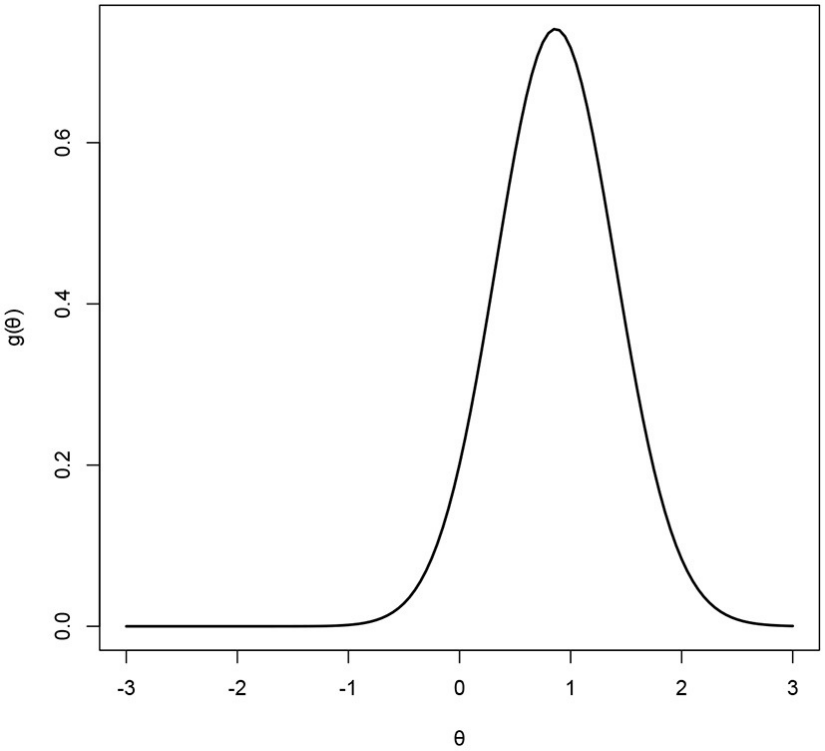
Estimated Ramsay-curves for the real data set.

The estimated latent-ability density curve of the RC-3PNO model is presented in Figure 6. It can be seen that the estimated latent trait density curve for this model has an obviously negatively skewed trend, which indicates that the math ability of these subjects is above the mean of the population. Therefore, the conventional methods assuming a normal distribution of latent abilities may result in negatively biased ability estimates. Table 8 shows the item parameter estimates for the 11 items of the PISA test form.

**Table 8:** Item parameter estimates under RC-3PNO model for the real data set.

**Item**	***a***	***b***	***c***	
1	0.23	0.00	0.12	
2	1.19	0.53	0.45	
3	0.88	−0.83	0.66	
4	0.39	0.76	0.92	
5	1.22	−0.09	0.55	
6	1.64	0.60	0.44	
7	1.89	−0.72	0.12	
8	1.17	−0.85	0.28	
9	1.56	−0.04	0.37	
10	1.24	−0.49	0.60	
11	1.26	−0.72	0.62	

## 7. Discussion

In real testing, the assumption of a normal distribution of latent abilities in IRT may be violated. For example, non-normality could result from the sampling of one or more distinct populations such as those with or without a “disorder.” In this case, the use of traditional algorithms, such as MML-EM, SAEM, and MCMC, in which the latent-ability distribution is restricted to normality, leads to severely biased parameter estimates (Woods, 2006, 2007; Azevedo et al., 2011; DeMars, 2012; Molenaar et al., 2012; Wall et al., 2015; Reise et al., 2018). Several methods are used to relax the normal assumption of latent trait distribution. For example, the EH method, log-linear smoothing, Davidian-curve IRT, and Ramsay-curve IRT, have been proposed to estimate the distribution of latent ability simultaneously with the item parameters. The Ramsay curve is flexible in that it can describe non-normal latent-ability distributions (Ramsay, 2000). To date, several RC-IRT models have been developed. For instance, the RC-2PL model (Woods and Thissen, 2006), RC-3PL model (Woods, 2008), and the logistic GRM incorporating a Ramsay curve (Monroe and Cai, 2014). However, the normal ogive model incorporating a Ramsay curve is rarely used due to the constraints that the normal ogive model itself requires for the integral calculation.

To fill the gap of estimating the normal ogive models in RC-IRT, we propose here an SAEM algorithm to estimate the RC-3PNO model with non-normal latent-ability distributions. In contrast to the traditional EM algorithm, the stochastic approximation step of the SAEM algorithm avoids the need for complex integral computation, and the M-step is straightforward to execute due to obtaining sufficient statistics of the MAP estimates for the item parameters in exponential family distributions, which greatly simplifies the computation and improves its efficiency. Compared with the MHRM algorithm, for exponential family distributions, the SAEM algorithm does not need differential calculations when the standard errors of estimates are not required; thus, the calculations of the SAEM algorithm are easier to execute. The estimates of the item parameters and the shape of the latent-ability density can be simultaneously obtained from this new algorithm. By introducing a Ramsay curve into the SAEM procedure, the new algorithm can be applied not only to a normal distribution of latent abilities but also to non-normal scenarios such as skewed and bimodal distributions.

Simulation study 1 investigated three model selection criteria to select the optimal *knots* and *degree* values in the B-spline functions of Ramsay curves. The choice of *knots* and *degree* had no noticeable influence on the bias and RMSE values of the item parameters. The results of simulation study 2 indicated that the proposed RC-SAEM algorithm generally performs better than the SAEM and MCMC algorithms when the true ability density is skewed or bimodal. Specifically, the RC-SAEM algorithm is obviously superior to the SAEM and MCMC algorithms according to the bias of item parameters in the RC-3PNO model when the true θ is skewed or bimodal. Although the RMSE values of item parameter estimates from the RC-SAEM algorithm are sometimes slightly larger than those of the SAEM in bimodal cases, especially for the *b* parameter, they are still within the acceptable range. Compared with the suggested sample size of 1,000 for the 3PL model used in RC-IRT (Woods, 2008), a sample size of 500 is large enough for the estimation of parameters in RC-3PNO with a test length of 15.

For the empirical example, according to the model selection criteria (AIC, BIC, and HQIC), the RC-3PNO model gives a better model fit than the 3PNO model. The shape of the estimated Ramsay curve indicates that the latent abilities of these examinees are mainly distributed at the higher level of the latent-ability continuum. In real testing, for binary responses influenced by guessing, although both the RC-3PL and RC-3PNO models serve as possible alternatives, the RC-3PNO model is suggested because the proposed SAEM algorithm avoids the need for calculations of the integral in the E-step and the derivatives in the M-step of the original EM algorithm, which greatly simplifies the computation. We suggest that the RC-3PNO model can be used to detect a non-normal shape in a latent trait distribution. In this case, our proposed RC-SAEM algorithm can also be adopted to simultaneously estimate the item parameters and the latent-ability density.

Several limitations and extensions of the proposed RC-SAEM algorithm need to be mentioned. First, the proposed RC-SAEM algorithm can be extended to other models, such as the GRM and the four-parameter normal ogive (4PNO) model (Culpepper, 2016). Second, a notable fact is that a multidimensional generalization of RC-IRT has not yet been developed. When such a development occurs, the proposed RC-SAEM algorithm can be extended to multidimensional models. Third, future research could compare the proposed RC-SAEM algorithm with other algorithms involving methods that relax the normality assumption of latent traits, such as DC-IRT and LLS. Fourth, the proposed RC-3PNO model together with the SAEM estimation algorithm could be investigated in other application domains, such as psychopathology measures involving evidently non-normal latent traits (e.g., borderline personality disorder and dark-triad traits) or medical fields (e.g., drug abuse). Finally, Kuhn and Lavielle (2004) have shown that the SAEM algorithm can also be used for estimating the asymptotic covariance matrix of the maximum-likelihood estimate, and this could be adopted in the RC-SAEM algorithm in the future.

## Data availability statement

Publicly available datasets were analyzed in this study. This data can be found at: https://www.oecd.org/pisa/.

## Author contributions

YC and JLu completed the writing of the article and original thoughts. JLi, YC, and JLu provided key technical support. YC provided the data. JZ, JLu, YC, and JLi completed the article revisions. NS and XM provided technical support. All authors contributed to the article and approved the submitted version.

## Funding

This work was supported by the National Natural Science Foundation of China (Grant No. 12001091), China Postdoctoral Science Foundations (Grant Nos. 2021M690587 and 2021T140108), and the Fundamental Research Funds for the Central Universities of China (Grant No. 2412020QD025).

## Conflict of interest

The authors declare that the research was conducted in the absence of any commercial or financial relationships that could be construed as a potential conflict of interest.

## Publisher's note

All claims expressed in this article are solely those of the authors and do not necessarily represent those of their affiliated organizations, or those of the publisher, the editors and the reviewers. Any product that may be evaluated in this article, or claim that may be made by its manufacturer, is not guaranteed or endorsed by the publisher.

## References

[B1] Akaike H. (1973). Maximum likelihood identification of Gaussian autoregressive moving average models. Biometrika 60, 255–265. 10.1093/biomet/60.2.255

[B2] Azevedo C. L. Bolfarine H. Andrade D. F. (2011). Bayesian inference for a skew-normal IRT model under the centred parameterization. Comput. Stat. Data Anal. 55, 353–365. 10.1016/j.csda.2010.05.003

[B3] Baker F. B. Kim S. H. (2004). Item Response Theory: Parameter Estimation Techniques, eds Pantula S. G. (North Carolina State University; CRC Press).

[B4] Béguin A. A. Glas C. A. (2001). MCMC estimation and some model-fit analysis of multidimensional IRT models. Psychometrika 66, 541–561. 10.1007/BF02296195

[B5] Birnbaum A. (1968). Some latent trait models and their use in inferring an examinee's ability, in Statistical Theories of Mental Test Scores, eds Lord F. M. Novick M. R. (Reading: MIT Press), 397–479.

[B6] Bock R. D. Aitkin M. (1981). Marginal maximum likelihood estimation of item parameters: application of an EM algorithm. Psychometrika 46, 443–459. 10.1007/BF02293801

[B7] Bock R. D. Lieberman M. (1970). Fitting a response model for dichotomously scored items. Psychometrika 35, 179–197. 10.1007/BF02291262

[B8] Cai L. (2010). High-dimensional exploratory item factor analysis by a Metropolis-Hastings Robbins-Monro algorithm. Psychometrika 75, 33–57. 10.1007/s11336-009-9136-x33528784

[B9] Camilli G. Geis E. (2019). Stochastic approximation EM for large-scale exploratory IRT factor analysis. Stat. Med. 38, 3997–4012. 10.1002/sim.8217 31267550

[B10] Casabianca J. M. Lewis C. (2015). IRT item parameter recovery with marginal maximum likelihood estimation using loglinear smoothing models. J. Educ. Behav. Stat. 40, 547–578. 10.3102/1076998615606112

[B11] Culpepper S. A. (2016). Revisiting the 4-parameter item response model: Bayesian estimation and application. Psychometrika 81, 1142–1163. 10.1007/s11336-015-9477-6 26400070

[B12] De Boor C. (1978). A Practical Guide to Splines. New York, NY: Springer-Verlag. 10.1007/978-1-4612-6333-3

[B13] Delyon B. Lavielle M. Moulines E. (1999). Convergence of a stochastic approximation version of the EM algorithm. Ann. Stat. 94–128. 10.1214/aos/1018031103

[B14] DeMars C. E. (2012). A comparison of limited-information and full-information methods in Mplus for estimating item response theory parameters for nonnormal populations. Struct. Equat. Model. Multidiscipl. J. 19, 610–632. 10.1080/10705511.2012.713272

[B15] Dempster A. P. Laird N. M. Rubin D. B. (1977). Maximum likelihood from incomplete data via the EM algorithm. J. R. Stat. Soc. Ser. B Stat Methodol. 39, 1–22. 10.1111/j.2517-6161.1977.tb01600.x

[B16] Geis E. (2019). Stochastic approximation EM for exploratory item factor analysis (Ph.D. thesis) Rutgers The State University of New Jersey, School of Graduate Studies, New Brunswick, NJ, United States.

[B17] Geyer C. J. (1994). On the convergence of Monte Carlo maximum likelihood calculations. J. R. Stat. Soc. Ser. B Stat. Methodol. 56, 261–274. 10.1111/j.2517-6161.1994.tb01976.x

[B18] Greig D. M. Porteous B. T. Seheult A. H. (1989). Exact maximum a posteriori estimation for binary images. J. R. Stat. Soc. Ser. B Methodol. 51, 271–279. 10.1111/j.2517-6161.1989.tb01764.x

[B19] Gu M. G. Zhu H. T. (2001). Maximum likelihood estimation for spatial models by Markov chain Monte Carlo stochastic approximation. J. R. Stat. Soc. Ser. B Methodol. 63, 339–355. 10.1111/1467-9868.00289

[B20] Hannan E. J. (1987). Rational transfer function approximation. Stat. Sci. 2, 135–151. 10.1214/ss/1177013343

[B21] Harwell M. R. Baker F. B. (1991). The use of prior distributions in marginalized Bayesian item parameter estimation: a didactic. Appl. Psychol. Meas. 15, 375–389. 10.1177/014662169101500409

[B22] Jank W. (2006). Implementing and diagnosing the stochastic approximation EM algorithm. J. Comput. Graph. Stat. 15, 803–829. 10.1198/106186006X157469

[B23] Kuhn E. Lavielle M. (2004). Coupling a stochastic approximation version of EM with an MCMC procedure. ESAIM Probabil. Stat. 8, 115–131. 10.1051/ps:2004007

[B24] Lee C. H. Gauvain J. L. (1996). “Bayesian adaptive learning and MAP estimation of HMM, in Automatic Speech and Speaker Recognition, eds Lee C. H. Soong F. K. Paliwal K. K. (Boston, MA: Springer), 83–107. 10.1007/978-1-4613-1367-0_4

[B25] Maydeu-Olivares A. Cai L. (2006). A cautionary note on using *G*^2^(dif) to assess relative model fit in categorical data analysis. Multivariate Behav. Res. 41, 55–64. 10.1207/s15327906mbr4101_4 26788894

[B26] Meng X. L. Rubin D. B. (1993). Maximum likelihood via the ECM algorithm: a general framework. Biometrika 80, 267–278. 10.1093/biomet/80.2.267

[B27] Mislevy R. J. (1986). Bayes modal estimation in item response models. Psychometrika 51, 177–195. 10.1007/BF02293979

[B28] Molenaar D. Dolan C. V. De Boeck P. (2012). The heteroscedastic graded response model with a skewed latent trait: testing statistical and substantive hypotheses related to skewed item category functions. Psychometrika 77, 455–478. 10.1007/s11336-012-9273-5 27519776

[B29] Monroe S. Cai L. (2014). Estimation of a Ramsay-Curve item response theory model by the Metropolis-Hastings Robbins-Monro algorithm. Educ. Psychol. Meas. 74, 343–369. 10.1177/0013164413499344

[B30] OECD. (2019). PISA 2018 Assessment and Analytical Framework. Paris: OECD Publishing. 10.1787/b25efab8-en

[B31] Ramsay J. O. (2000). Differential equation models for statistical functions. Can. J. Stat. 28, 225–240. 10.2307/3315975

[B32] Reise S. P. Revicki D. A. (2014). Handbook of Item Response Theory Modeling: Applications to Typical Performance Assessment. New York, NY: Taylor and Francis. 10.4324/9781315736013

[B33] Reise S. P. Rodriguez A. Spritzer K. L. Hays R. D. (2018). Alternative approaches to addressing non-normal distributions in the application of IRT models to personality measures. J. Pers. Assess. 39, 363–374. 10.1080/00223891.2017.1381969 29087217 PMC6252010

[B34] Robbins H. Monro S. (1951). A stochastic approximation method. Ann. Math. Stat. 22, 400–407. 10.1214/aoms/1177729586

[B35] Samejima F. (1969). Estimation of latent ability using a response pattern of graded scores. Psychometrika 34 (Suppl. 1), 1–97. 10.1007/BF0337216027519776

[B36] Schwarz G. (1978). Estimating the dimension of a model. Ann. Stat. 6, 461–464. 10.1214/aos/1176344136

[B37] Wall M. M. Park J. Y. Moustaki I. (2015). IRT modeling in the presence of zero-inflation with application to psychiatric disorder severity. Appl. Psychol. Meas. 39, 583–597. 10.1177/0146621615588184 29881029 PMC5978495

[B38] Wang C. Su S. Weiss D. J. (2018). Robustness of parameter estimation to assumptions of normality in the multidimensional graded response model. Multivariate Behav. Res. 53, 403–418. 10.1080/00273171.2018.1455572 29624093

[B39] Wei G. C. G. Tanner M. A. (1990). A Monte Carlo implementation of the EM algorithm and the poor man's data augmentation algorithms. J. Am. Stat. Assoc. 85, 699–704. 10.1080/01621459.1990.10474930

[B40] Woods C. M. (2006). Ramsay-curve item response theory (RC-IRT) to detect and correct for nonnormal latent variables. Psychol. Methods 11, 253–270. 10.1037/1082-989X.11.3.253 16953704

[B41] Woods C. M. (2007). Ramsay-curve IRT for Likert-type data. Appl. Psychol. Meas. 31, 195–212. 10.1177/0146621606291567

[B42] Woods C. M. (2008). Ramsay-curve item response theory for the three-parameter logistic item response model. Appl. Psychol. Meas. 32, 447–465. 10.1177/0146621607308014

[B43] Woods C. M. Lin N. (2009). Item response theory with estimation of the latent density using Davidian curves. Appl. Psychol. Measure. 33, 102–117. 10.1177/0146621608319512

[B44] Woods C. M. Thissen D. (2006). Item response theory with estimation of the latent population distribution using spline-based densities. Psychometrika 71, 281–301. 10.1007/s11336-004-1175-8 28197961

[B45] Zhang D. Davidian M. (2001). Linear mixed models with flexible distributions of random effects for longitudinal data. Biometrics 57, 795–802. 10.1111/j.0006-341X.2001.00795.x 11550930

