# Supplementary material for: A stochastic approximation expectation maximization algorithm for estimating Ramsay-curve three-parameter normal ogive model with non-normal latent trait distributions
Source: Front Psychol. 2022 Nov 24;13:971126. doi: 10.3389/fpsyg.2022.971126 (PMC9730697; doi:10.3389/fpsyg.2022.971126)
Supplement: Supplementary file 1 [file Presentation_1.pdf]

Appendix

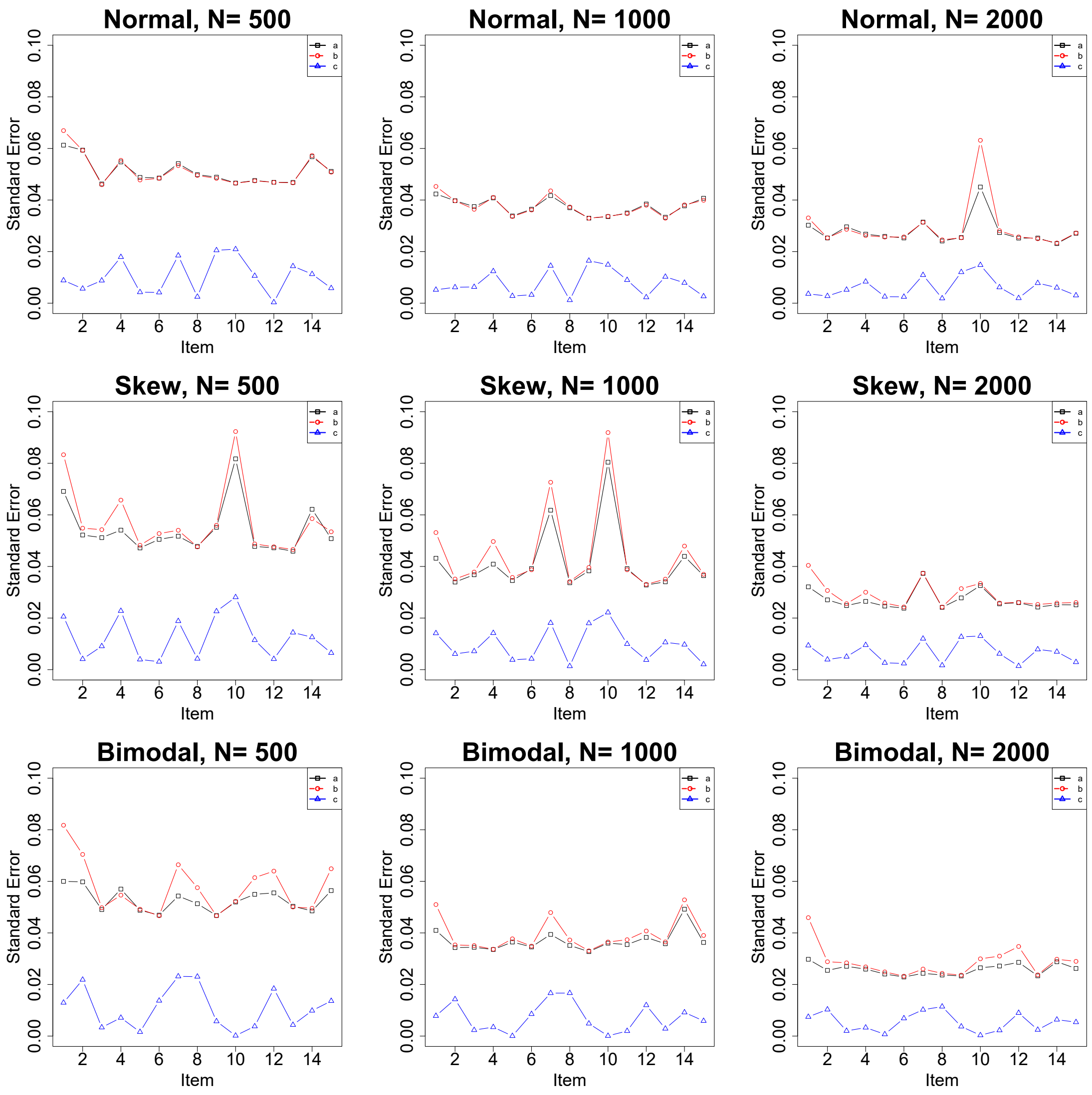

Figure A1: The standard error of item parameters using the RC-SAEM algorithm when test length is 15 in simulation study 2.

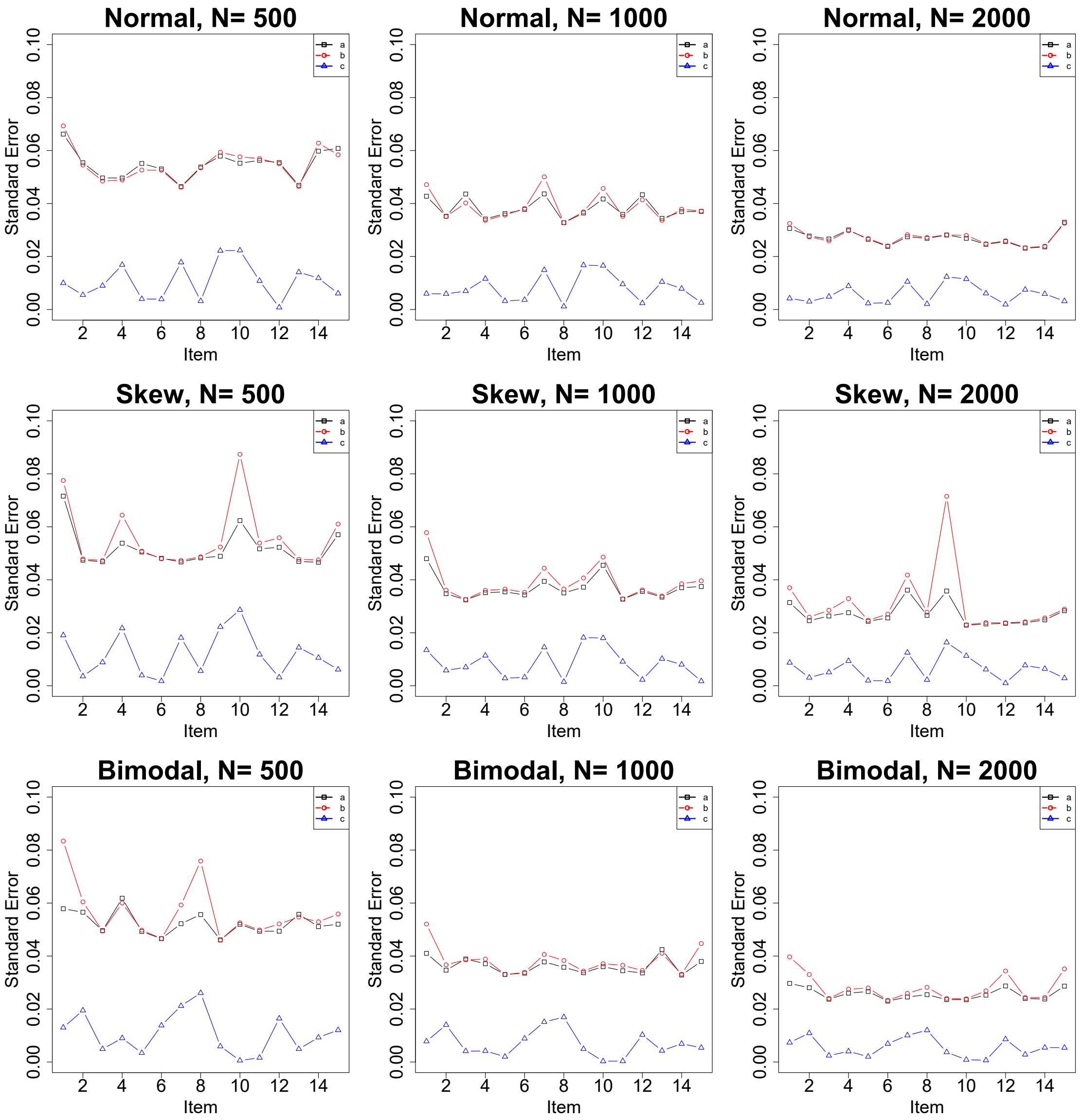

Figure A2: The standard error of item parameters using the SAEM algorithm when test length is 15 in simulation study 2.

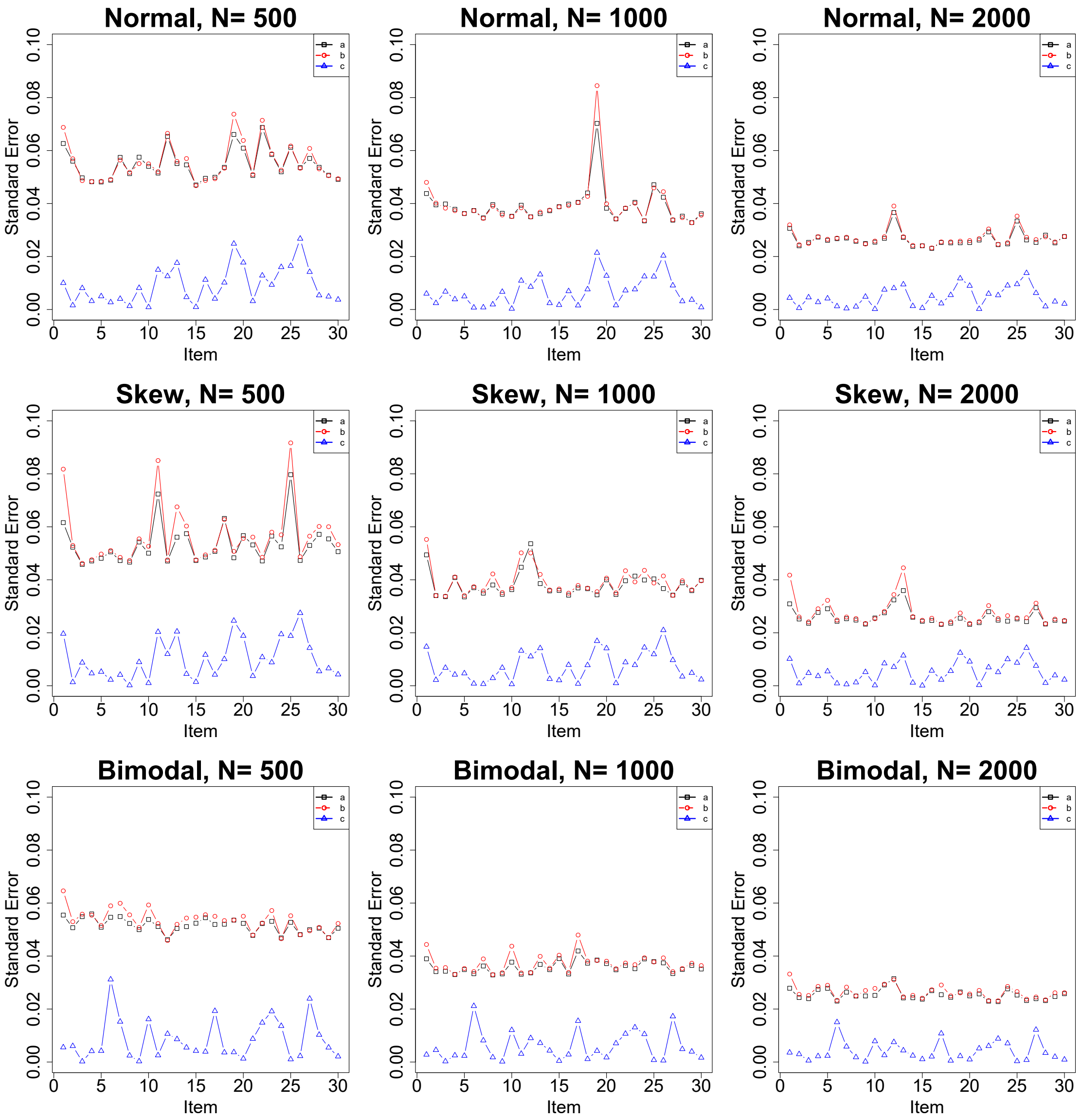

Figure A3: The standard error of item parameters using the RC-SAEM algorithm when test length is 30 in simulation study 2.

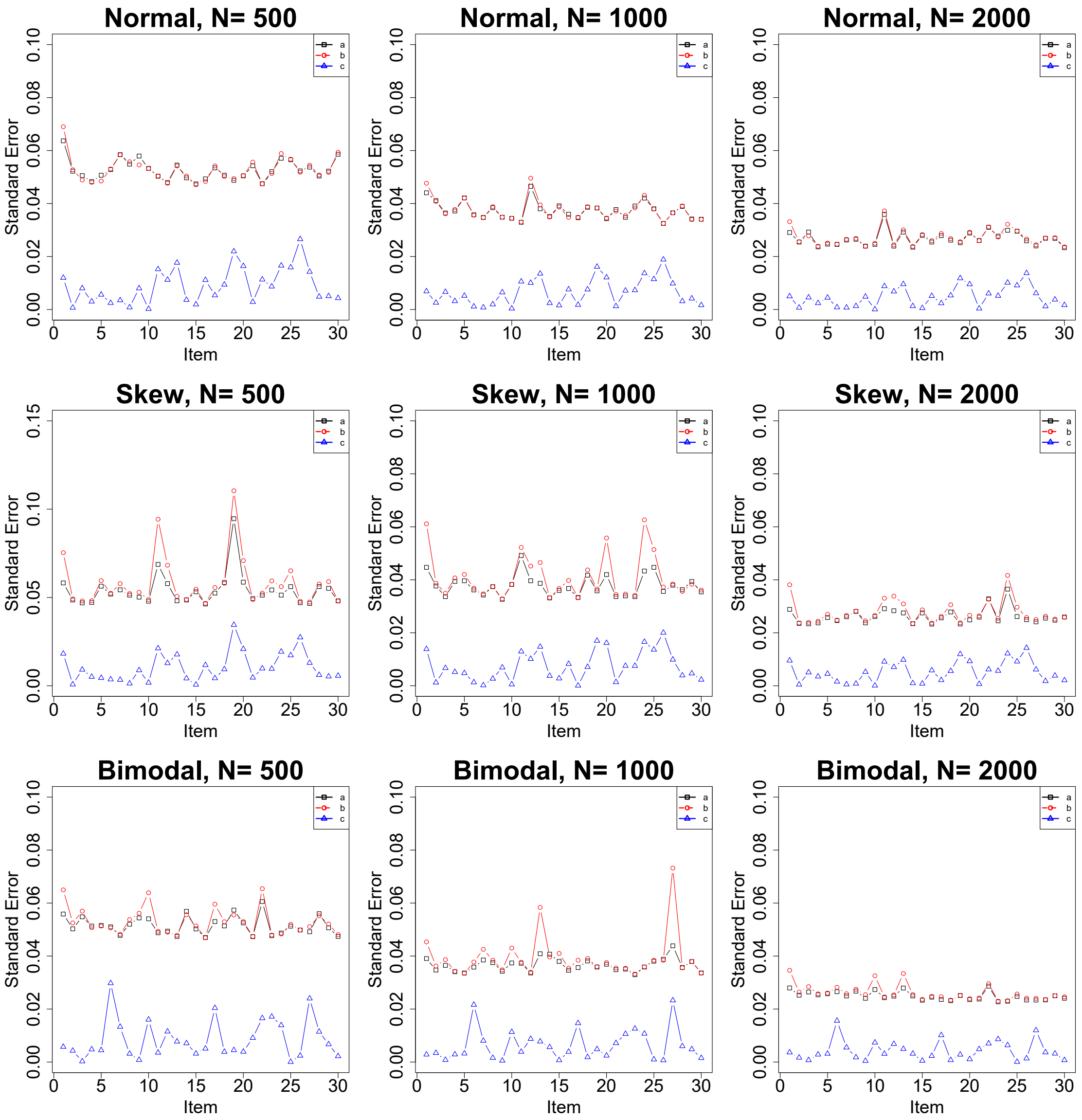

Figure A4: The standard error of item parameters using the SAEM algorithm when test length is 30 in simulation study 2.
